# Genetic diversity of *Diaphorina citri* (Hemiptera: Liviidae) unravels phylogeographic structure and invasion history of eastern African populations

**DOI:** 10.1002/ece3.9090

**Published:** 2022-07-17

**Authors:** Inusa Jacob Ajene, Fathiya Mbarak Khamis, Barbara van Asch, Gerhard Pietersen, Nurhussen Seid, Anne Wambui Wairimu, Fidelis Levi Ombura, Komivi Senyo Akutse, Mamoudou Sétamou, Sevgan Subramanian, Samira Mohammed, Sunday Ekesi

**Affiliations:** ^1^ Department of Crop Protection Faculty of Agriculture Ahmadu Bello University Zaria Nigeria; ^2^ International Center of Insect Physiology and Ecology Nairobi Kenya; ^3^ Department of Genetics Stellenbosch University Stellenbosch South Africa; ^4^ Ethiopian Institute of Agricultural Research Addis Ababa Ethiopia; ^5^ Texas A&M University Kingsville Citrus Centre Weslaco Texas USA

**Keywords:** Asian citrus psyllid, eastern Africa, microsatellites, mitogenome

## Abstract

The Asian citrus psyllid (*Diaphorina citri* Kuwayama) is a key pest of *Citrus* sp. worldwide, as it acts as a vector for *Candidatus* Liberibacter asiaticus, the bacterial pathogen that causes citrus Huanglongbing. *Diaphorina citri* has been reported in Kenya, Tanzania, and more recently in Ethiopia. This study assessed the genetic diversity and phylogeographic structure of the pest to gain insights into the potential sources of its introduction into Africa. Population structure and differentiation of *D. citri* populations from China, Ethiopia, Kenya, Tanzania, and the USA were assessed using 10 microsatellite loci. Additionally, five new complete mitogenomes of *D. citri* collected in China, Ethiopia, Kenya, Tanzania, and the USA were analyzed in the context of publicly available sequences. Genotype data grouped the *D. citri* populations from Kenya and Tanzania in one cluster, and those from Ethiopia formed a separate cluster. The two genetic clusters inferred from genotype data were congruent with mitochondrial sequence data. The mitogenomes from Kenya/Tanzania/China had 99.0% similarity, and the Ethiopia/USA had 99.9% similarity. In conclusion, *D. citri* populations in eastern Africa have different sources, as the Kenyan and Tanzanian populations probably originated from southeastern Asia, while the Ethiopian population most probably originated from the Americas.

## Graphical abstract

The Asian citrus psyllid (Diaphorina citri Kuwayama) is a key pest of Citrus sp. worldwide, as it acts as a vector for Candidatus Liberibacter asiaticus, the bacterial pathogen which causes citrus Huanglongbing. This study assessed the genetic diversity and phylogeographic structure of the pest to gain insights into the potential sources of its introduction into Africa. The research showed, D. citri populations in eastern Africa have different sources, as the Kenyan and Tanzanian populations probably originated from China while the Ethiopian population most probably originated from the USA.

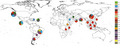

## 1 INTRODUCTION

*Diaphorina citri Kuwayama* (Hemiptera: Liviidae), commonly known as the Asian citrus psyllid, is currently found in tropical and subtropical Asia, Afghanistan, Saudi Arabia, Reunion, Mauritius, parts of South and Central America, the United States of America, Mexico and the Caribbean (Grafton‐Cardwell et al., 2006), Tanzania (Shimwela et al., 2016), Kenya (Rwomushana et al., 2017) and Ethiopia (Ajene et al., 2020a) in East Africa, and Nigeria in West Africa (Oke et al., 2020). The feeding activities of *D. citri* cause direct damage to plants such as curling of the leaves (Grafton‐Cardwell et al., 2006). Heavy citrus tree infestation by *D. citri* hampers the normal development of flush shoots, thus making them more likely to break off. Additionally, the production of honeydew by the insect on the plant leads to sooty mold growth, which reduces the photosynthetic activity and productivity of trees. The most significant impact of *D. citri* on citrus, however, is the transmission of the phloem‐limited bacterium *Candidatus* Liberibacter asiaticus (Las), the causal pathogen of Huanglongbing (Bové, 2006). Las is closely related to *Candidatus* Liberibacter americanus (Lam) and *Candidatus* Liberibacter africanus (Laf), a bacterium that is transmitted by *Trioza erytreae* (Hemiptera: Triozidae), a pest widespread in East and Southern African highlands (Rasowo et al., 2019). Las has been reported in Asia, and North and South America since 2004 (Grafton‐Cardwell et al., 2013; Halbert & Manjunath, 2004; Hall et al., 2013). In Africa, Las was reported for the first time in Ethiopia in 2010 (Saponari et al., 2010) but its primary vector *D. citri* was not detected until 2020 (Ajene et al., 2020a). *Diaphorina citri* has been shown, albeit experimentally, to transmit Laf in laboratory conditions (Massonie et al., 1976). Given the current context, the ongoing invasion of Africa by *D. citri* is a significant threat to citrus production on the continent.

Assessments of genetic diversity are useful in a wide range of scientific fields, including animal and plant breeding, evolution, conservation, pest control, and taxonomy (Wangari et al., 2013; Wu et al., 1999).

Knowledge on the genetic diversity of citrus psyllids across different geographical areas is useful for the assessment and management of risk and spread (Jantasorn et al., 2012). Different symptoms of citrus greening disease in various host species may be affected by the genetic diversity of the bacterial pathogen; for instance, symptoms of greening caused by *Candidatus* Liberibacter africanus are less severe than observed in trees infected by the subspecies *Candidatus* Liberibacter africanus subspecies clausenae (Ajene et al., 2020b). Furthermore, the movement and distribution of the insect vector in some geographical regions may also be affected by its genetic diversity (Jantasorn et al., 2012). The potential climatic suitability of *D. citri* in citrus growing areas in Africa poses a risk of rapid spread of citrus greening across the continent (Shimwela et al., 2016).

The genetic diversity of *D. citri* populations from different geographical regions has been assessed using mitochondrial cytochrome oxidase 1 (COI) sequences (De León et al., 2011; Boykin et al., 2012; Guidolin & Consoli, 2013; Lashkari et al., 2014), and showed that the presence of *D. citri* in the Americas resulted from two separate introductions (De León et al., 2011). Furthermore, two major mitochondrial groups have been identified in southwestern and southeastern Asia, and allowed for tracing the *D. citri* invasion of Florida, Texas, and Mexico to southwestern Asia (Boykin et al., 2012). The mitochondrial lineages of *D. citri* in Kenya and La Réunion showed a probable link between *D. citri* from Kenya and southeastern Asia (Wang et al., 2021). Also, a comparison of the microbiome and key endosymbionts of *D. citri* showed a close similarity between psyllids from China and those found in Kenya and Tanzania (Ajene et al., 2020c).

Simple sequence repeat markers, also known as microsatellites, are an important tool for determining genetic variation in populations (Sajib et al., 2012; Powell et al., 1996). Microsatellites are commonly used in genetic diversity studies, gene mapping (Ma et al., 2011; Sajib et al., 2012; Zhang et al., 2007), and genetic fingerprinting (Ma et al., 2011; Sajib et al., 2012; Xiao et al., 2006) because they are biparentally transmitted and generally highly polymorphic, and statistical tools for genotype data analyses are well developed and widely available. Microsatellite markers are employed to track possible geographical origin(s) of pest species (Boykin et al., 2007; Fraimout et al., 2015; Khamis et al., 2009; Ruíz‐Rivero et al., 2021), and in our case, the markers have been used to infer the invasion pattern of introduction and spread of *D. citri* into Africa. This information can be instrumental in the development of appropriate management strategies and monitoring of expansions of the geographic range of this agricultural pest of economic importance. The notable rapid spread of *D. citri* in Tanzania and Kenya and its recent detection in Ethiopia indicate a high risk of invasion and establishment of the pest in other regions of eastern and Southern Africa. Therefore, gaining insights into the population structure and genetic diversity of African *D. citri* populations using microsatellite markers is relevant and opportune.

This study aimed to provide baseline genetic data for understanding the population dynamics and genetic structure of *D. citri* in eastern Africa, and to gain insights into the geographic origin of the invasion.

## 2 METHODS

### 2.1 Sample collection and DNA extraction

Field surveys were carried out in citrus orchards and backyard gardens in Ethiopia, Kenya, and Tanzania from March 2017 to December 2019. Specimens of *D. citri* were aspirated from host trees and stored individually in 96% ethanol until DNA extraction. Geographic positioning system (GPS) coordinates of the sampling sites were recorded for each location using a Garmin eTrex20 instrument (GARMIN, USA). A number of *D. citri* specimens from the USA (Kingsville Citrus Centre, Texas A&M University) and China (Fuzhou, Fujian Province) were collected by local collaborators and preserved in ethanol. A total of 270 specimens from 10 locations (one site in China, one site in Ethiopia, four sites in Kenya, three sites in Tanzania, and one in the USA) (Table 1) were used in the analyses.

**TABLE 1:** Collection data of *Diaphorina citri* populations used in this study

Country	Collection site	Code	Latitude	Longitude	*n*
China	Fuzhou	CHN	26.079	119.297	10
Ethiopia	Goshuha	ETH	11.764	39.5917	20
Kenya	Awasi	AWA	0.167	35.0844	30
	Koitamburot	KOT	−0.207	35.1926	30
	Lungalunga	LUN	−4.563	39.1221	30
	Soin	SOI	0.5412	35.1846	30
Tanzania	Mafiga	MAF	−5.22	37.6593	30
	Mikese	MIK	−4.935	39.1259	30
	Mlali	MLA	−6.997	37.5705	30
USA	Texas	USA	30.015	−96.3425	30

*Note: n* = number of samples per site.

Total DNA was extracted from individual specimens using the Isolate II Genomic DNA kit (Bioline, London, UK), following the manufacturer's protocol. DNA extracts were checked for quality and concentration using a Nanodrop 2000/2000c Spectrophotometer (Thermo Fisher Scientific, Wilmington, USA). DNA extracts within the A_260 nm_/A_280 nm_ ratio range of 1.8 to 2.0 were eluted to a final volume of 50 μl and used for downstream analyses.

### 2.2 Microsatellite genotyping

Ten to 30 individuals from each of the 10 locations (Table 1) were genotyped using ten microsatellite markers (Dcoi01, Dcoi02, Dcoi03, Dcoi04, Dcoi05, Dcoi07, Dcoi09, Dcoi10, Dcoi11, and Dcoi12) (Boykin et al., 2012). The forward primer of each pair was fluorescently labeled for automatic detection in capillary electrophoresis (see Table S1). Multiplex PCR amplifications were performed in a total reaction volume of 20 μl containing 5X MyTaq Reaction Buffer (5 mM dNTPs, 15 mM MgCl_2,_ stabilizers, and enhancers) (Bioline, London, UK), 0.5 pmol μl^−1^ of each primer, 0.5 mM MgCl_2_, 0.0625 U μl^−1^ of MyTaq DNA polymerase, and 15 ng μl^−1^ of DNA template. Cycling conditions were as follows: initial denaturation at 95°C for 2 min, 40 cycles of 95°C for 30 s, annealing temperature (Ta) for 45 s, 72°C for 1 min, and a final extension at 72°C for 10 min. PCR amplifications were run on a Mastercycler gradient Nexus thermal cycler (Eppendorf, Hamburg, Germany). Negative controls were included in all reactions. PCR products were run on an ABI PRISM 3730 DNA (Applied Biosystems, Waltham, MA, USA) analyzer with HiDi Formamide as single‐stranded DNA stabilizer, GeneScan 500 ROX (Applied Biosystems) as internal size standard, and Pop‐7 (Applied Biosystems) as sieving matrix. Allele calling was performed with ABI PRISM GeneMarker software version 3.0.1 (Softgenetics).

### 2.3 Marker summary statistics and intrapopulation genetic diversity

Microsatellite data were prepared for analyses using the MS Tools add‐in for Microsoft Excel (Park, 2001). Assessment of population genetic parameters was performed in the GenAlEx Add‐in for Microsoft Excel (Peakall & Smouse, 2012). Genetic differentiation was estimated using standard genetic distances. Allele frequencies by population (AFP), heterozygosity, F‐statistics, and polymorphism by population (HFP), allelic pattern graph (APT), private alleles by population (PAS), and samples with more than one private allele (PAL) were calculated in GenAlEx.

### 2.4 Population structure analysis

An analysis of molecular variance (AMOVA) for testing different population groupings to determine whether the metapopulation is in panmixia and the component of genetic diversity attributable to variance between and within populations from the different countries, and F_ST_ statistics were performed in Arlequin v3.5.2.2 (Excoffier & Lischer, 2010). Significance for the AMOVA was tested using 10,000 permutations. Exact tests for linkage disequilibrium and deviation from the Hardy–Weinberg equilibrium were conducted using the web version of GENEPOP (Raymond & Rousset 1995). Null alleles, stutters, and allelic dropout analysis were estimated using Micro‐Checker v. 2.2.3 (Van Oosterhout et al., 2004).

Two methods were used to infer the clustering of the 10 populations: Bayesian clustering and multivariate discriminant analysis of principal components (DAPC). The Bayesian approach was implemented in Structure 2.2 (Pritchard et al., 2000), with K values set from 1 to 10 to determine the most likely number of clusters present in the dataset. The simulations were run using the admixture model without prior population information. The length of the initial burn‐in period was set to 100,000 iterations followed by a run of 1000,000 Markov chain Monte Carlo (MCMC) repetitions, replicated 10 times to ensure convergence on parameters and likelihood values. The most likely number of subpopulations (K) in the dataset was identified with the Med‐ and Mean K methods (Puechmaille, 2016), comprising of four estimators: median of means (MedMeaK), maximum of means (MaxMeaK), median of medians (MedMedK), and maximum of medians (MaxMedK) where a subpopulation is assigned to a cluster based on its arithmetic mean (for MedMeaK and MaxMeaK) or its median (for MedMedK and MaxMedK). This method takes into account and corrects for uneven sampling. The most likely number of K that explained the structure in data was selected using the online resource STRUCTURE SELECTOR (Li & Liu, 2018), which calculates the estimators aid in the selection and visualization of the optimal K. The multivariate clustering approach was implemented using adegenet (Jombart, 2008) and poppr (Kamvar et al., 2014) packages in R version 3.5.1 with R‐Studio (R Development Core Team, 2008). GeneClass 2.0 (Piry et al., 2004) was used to assign or exclude reference populations as possible origins of individuals, based on the multilocus genotypes. The Nei's standard (1972) criterion was used for assignment of migrant populations. The Monte Carlo resampling method (Paetkau et al., 2004) based on 10,000 simulated genotypes for each population and on a threshold probability value of 0.05 was used to identify the accurate exclusion/inclusion critical values to assign the individuals in the populations. Data format conversions for the various software programs were performed with CONVERT version 1.31 (Glaubitz, 2004).

### 2.5 Mitogenome sequencing, assembly, and annotation

One adult specimen per country (China, Ethiopia, Kenya, Tanzania, and the USA) was used for next‐generation sequencing (NGS) for the recovery of complete mitochondrial genome sequences. Total DNA from five specimens was sequenced separately (whole genome) by Macrogen Inc., Europe (the Netherlands) using an Illumina MiSeq platform (Illumina, San Diego, CA, USA). Mapping and assembly of the mitogenome sequences were performed in Geneious Prime version 2019.0.4. (https://www.geneious.com) (Kearse et al., 2012). The NGS reads for each sample were mapped and assembled using a mitochondrial genome sequence of *D. citri* available on GenBank as reference (KU647697). The open reading frames of protein‐coding genes (PCGs) were identified with the invertebrate mitochondrial genetic code. The mapped sequences were aligned to the reference mitogenome and checked for stop codons and frameshift mutations using Geneious Prime. Transfer RNAs (tRNAs) were predicted with ARWEN v.1.2 (http://130.235.244.92/ARWEN/) (Laslett & Canbäck, 2008) using the default composite metazoan mitochondrial code. Ribosomal RNAs and the AT‐rich region were annotated by comparison with the reference sequence. Overlapping regions and intergenic spacers were counted manually. Nucleotide composition and AT and GC skews [AT skew = (A−T)/(A+T); GC skew = (G−C)/(G+C)] were calculated using Geneious Prime.

### 2.6 Comparison of mitogenome sequences

To assess genetic divergence and identify single nucleotide polymorphisms (SNPs) within the PCGs, rRNAs, and tRNAs among the *D. citri* mitogenomes, multiple sequence alignments of the sequences obtained in this study and 31 publicly available sequences (see Table S2) were performed using the MAFFT algorithm (Katoh et al., 2013) available in Geneious Prime. Genetic distances among all sequences were calculated using nucleotide pairwise distances (p‐distances) in MEGA X (Kumar et al., 2018) under the Kimura 2‐parameter model (Kimura, 1980). Genetic distances among the mitogenomes were represented with multidimensional scaling analysis using the “cmdscale” function in R version 3.5.1 (R Development Core Team, 2008) on the genetic distance matrix to generate the plot for principal coordinate analysis (PCoA).

### 2.7 Phylogeographic structure of *D. citri*

The phylogeographic structure of *D. citri* was assessed using maximum‐likelihood (ML) trees and median‐joining (MJ) networks. The five COI and 16S rRNA sequences extracted from the complete mitogenome generated in this study and other COI (*n* = 573) and 16S rRNA (*n* = 249) sequences available on GenBank (see Table S3) were used to generate separate ML trees. Multiple sequence alignments were performed using MAFFT and used to construct the MJ network with Network software v5 (http://www.fluxus‐engineering.com/sharenet.htm), under the default settings (Bandelt et al., 1999). Genetic divergences among the COI haplogroups displayed in the MJ network were calculated as pairwise distances (p‐distances) using MEGA X. ML trees constructed in MEGA X using the Tamura 3‐parameter model (T92) (Tamura, 1992) with *Bactericera cockerelli* (Šulc) (Hemiptera: Triozidae) and *Heteropsylla cubana* Crawford (Hemiptera: Psyllidae) as outgroups were used as alternative displays of the phylogenetic relationships among the sequences. Nodal support for ML trees was performed using 1000 bootstrap replicates. Multidimensional scaling analysis was carried out using the “cmdscale” function in R on the genetic distance matrix to generate the plot for principal coordinate analysis (PCoA). The R code is publicly available at https://github.com/InusaJacob/Principal‐Coordinate‐axes.

## 3 RESULTS

### 3.1 Genetic diversity of *Diaphorina citri*

These results are based on 10 microsatellite loci genotyped in 270 psyllids across 10 populations. The population from Lungalunga, Kenya, had the highest number of different alleles (3.30), while the population from Ethiopia had the highest number of effective alleles (2.54). All populations had a negative fixation index, which was highest for the population from Soin, Kenya. The population from Mlali, Tanzania, had the highest observed heterozygosity, while the population from Mikese, Tanzania, had the lowest. All populations had higher observed heterozygosity than expected heterozygosity (see Table S4).

Pairwise FST values were generally low and ranged from 0.012 to 0.305, and some of the lowest FST values were nonsignificant (Table 2). No out‐of‐range high values were observed between any of the African populations and China, or between the African populations and the USA. Eastern African populations had low differentiation. FST values were nonsignificant among populations from western Kenya (Soin, Awasi, and Koitamburot), but there was significant divergence between these locations and Lungalunga in coastal Kenya.

**TABLE 2:** Pairwise FST divergence between 10 different geographical populations of *Diaphorina citri*

	Population	China	Ethiopia	Kenya				Tanzania			USA
		Fuzhou	Goshuha	Awasi	Koitamburot	Lungalunga	Soin	Mafiga	Mikese	Mlali	Texas
China	Fuzhou	–	0.005	0.001	0.001	0.005	0	0.007	0.001	0	0
Ethiopia	Goshuha	0.059	–	0.001	0.001	0.012	0	0.007	0.001	0.01	0
Kenya	Awasi	0.194	0.161	–	0.017	0.001	0.02	0.001	0.001	0	0
	Koitamburot	0.138	0.125	0.023 _ns_	–	0.001	0.04	0.001	0.001	0	0
	Lungalunga	0.057	0.032 _ns_	0.107	0.078	–	0	0.076	0.002	0.02	0
	Soin	0.134	0.114	0.022 _ns_	0.012 _ns_	0.079	–	0	0	0	0
Tanzania	Mafiga	0.053	0.041	0.113	0.08	0.013 _ns_	0.086	–	0.002	0.01	0
	Mikese	0.145	0.1	0.305	0.26	0.07	0.255	0.082	–	0	0
	Mlali	0.107	0.056	0.207	0.179	0.038 _ns_	0.18	0.036 _ns_	0.051	–	0
USA	Texas	0.128	0.083	0.28	0.248	0.134	0.23	0.139	0.189	0.12	–

*Note:* FST values are shown below the diagonal. Probability, P (rand ≥ data), based on 999 permutations is shown above diagonal. Ns—not significant from zero (*p* < .05).

### 3.2 Population structure

The AMOVA performed across all groups revealed high variance within the populations (80.5%) and low variance among populations (19.5%), indicating that the populations are in panmixia. The AMOVA performed on large‐scale region groups (Africa separated from all other populations) showed that genetic differentiation among groups accounted for 9.8% of the total variance. All fixation indices, including FCT, FSC, FST, FIS, and FIT, were highly significant (*p* < .01) (Table 3).

**TABLE 3:** Analysis of molecular variance (AMOVA) of *Diaphorina citri* populations from China, Ethiopia, Kenya, Tanzania, and the USA

Hypothesis tested	Source of variation	Sum of squares	Variance component	Percentage variation (%)	Fixation index
Panmixia	Among populations	87.505	0.3802	19.505	*F*_ST_ = 0.16923
	Within populations	336.439	1.569	80.495	
Intercontinent	Among groups	57.123	0.2304	11.554	*F*_CT_ = 0.11554
	Among populations within groups	30.382	0.1949	9.773	*F*_SC_ = 0.11050
	Within groups	336.439	1.569	78.673	*F*_ST_ = 0.21327

**FIGURE 1:**
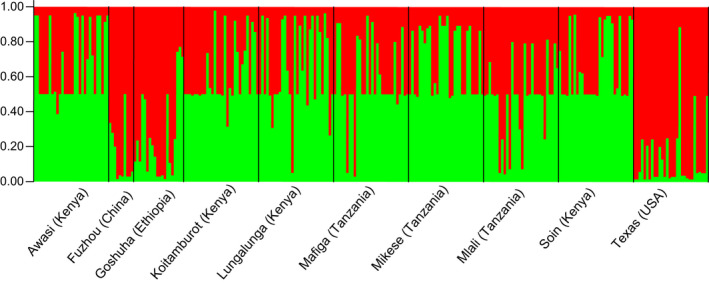
Structure bar plot showing the shared ancestry of *Diaphorina citri* (*n* = 270) in two hypothetical clusters (K1‐K2) based on 10 microsatellite genotypes. Black vertical lines separate collection sites

**FIGURE 2:**
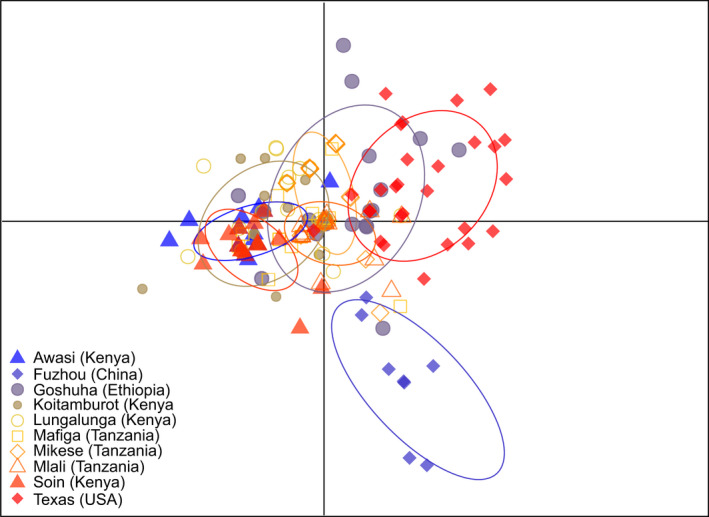
Multivariate analyses of population structure of 270 *Diaphorina citri* individuals from five countries (China, Ethiopia, Kenya, Tanzania, and the USA) using discriminate analysis of principal components

STRUCTURE analyses showed that the maximum value for the estimated likelihood of K was found at *K* = 2 (see Figure S1 in Appendix S1). The average values of ancestry probabilities (*Q*) for each population in the two clusters showed that the psyllids from Texas (USA) had the highest ancestry in one cluster (*Q* = 0.863), while the psyllids from Mikese (Tanzania) had the highest ancestry in the second cluster (*Q* = 0.708). Among African populations, the psyllids from Kenya and Tanzania grouped in one cluster, while those from Ethiopia were in the second cluster (see Table S5). Visualization of cluster membership coefficients showed that psyllids from China, Ethiopia, and the USA formed one cluster (red), while the psyllids from Kenya and Tanzania formed another cluster (green) (Figure 1). DAPC showed a similar pattern of similarity between the Kenyan and the Tanzanian populations, while the psyllids from Ethiopia clustered with those from the USA (Figure 2).

### 3.3 Assignment rate

**FIGURE 3:**
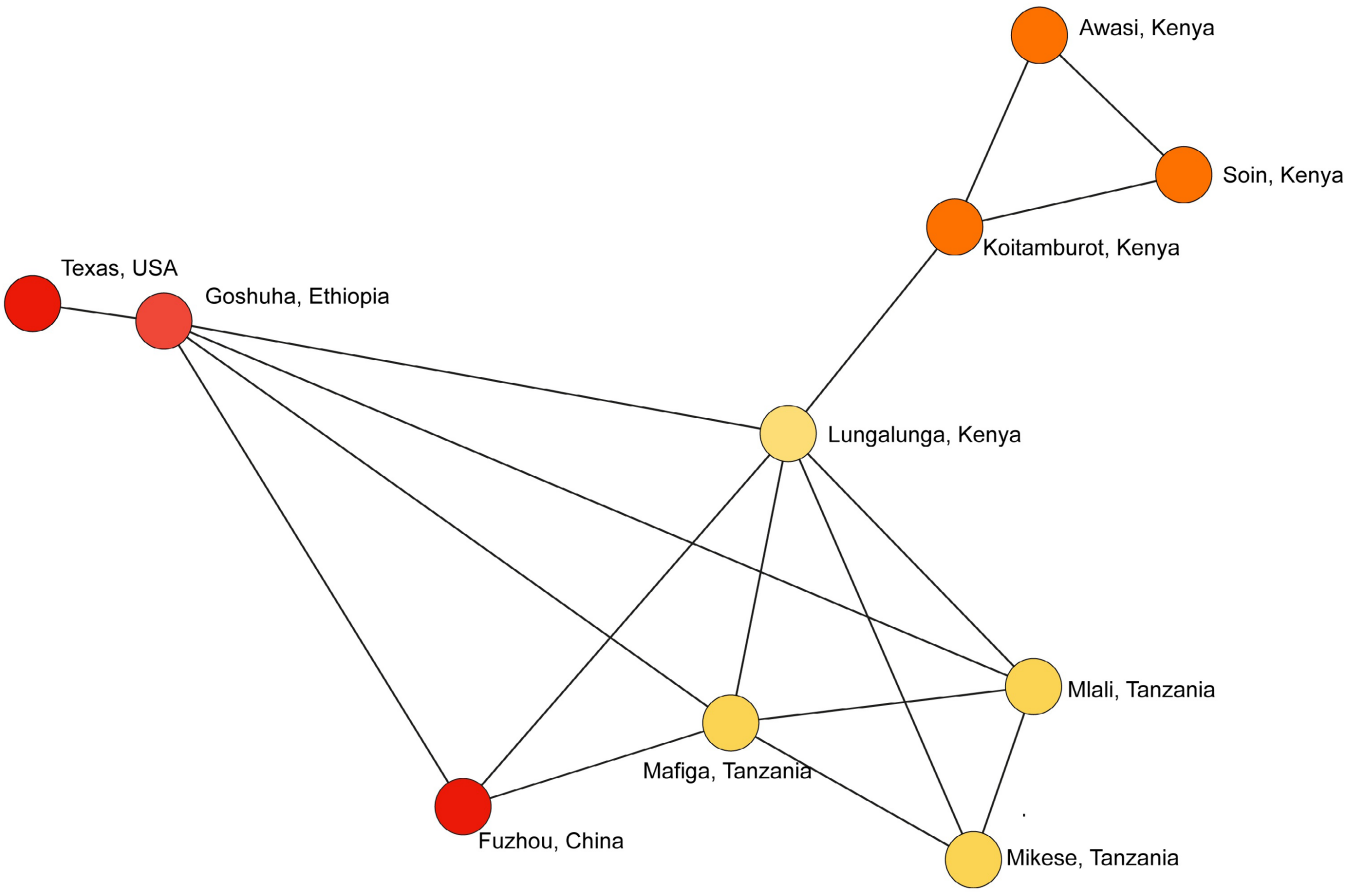
Minimum spanning tree network of 270 *Diaphorina citri* individuals from 10 geographic locations based on F_ST_ distance. The lines between circles indicate the similarity between profiles. Nodes are colored based on proportion of shared genotypes

Migration rate estimate values above 0.500 imply restricted gene flow, whereas values less than 0.500 suggest moderate‐to‐high gene flow. The migration rates from the Goshuha and Texas populations to other populations were all above 0.500. The migration rates among the Kenyan populations showed restricted gene flow between Lungalunga and the other Kenyan populations (average = 0.997). Interestingly, the average estimated migration rates between Lungalunga and the Tanzanian populations (0.210) are indicative of high gene flow (Table 4). The migration pattern displayed as a spanning tree network showed that Lungalunga was more closely linked to the Tanzanian populations than to the other Kenyan populations (Figure 3).

**TABLE 4:** Mean assignment rate of *Diaphorina citri* individuals into source populations (rows) and aim populations (columns) as calculated using Nei's standard distance in GENECLASS 2.0

	Fuzhou	Goshuha	Soin	Awasi	Koitamburot	Lungalunga	Mafiga	Mikese	Mlali	Texas	Assigned migrant
Fuzhou	0	1.032	1.437	0.982	1.224	0.588	**0.372**	0.69	0.623	0.992	Mafiga
Goshuha	1.032	0	0.751	0.952	1.103	0.584	0.817	0.952	0.613	**0.099**	Texas
Soin	1.437	0.751	0	0.28	0.399	0.917	0.92	2.478	1.71	0.94	Awasi
Awasi	0.982	0.952	0.28	0	0.63	1.14	1.01	2.7	1.74	0.94	Soin
Koitamburot	1.224	1.103	0.399	0.63	0	0.875	0.532	1.767	1.348	1.711	Soin
Lungalunga	0.588	0.584	0.917	1.14	0.875	0	0.297	0.135	0.199	0.592	Mikese
Mafiga	0.372	0.817	0.92	1.01	0.532	0.297	0	0.494	0.227	0.876	Mlali
Mikese	0.69	0.952	2.478	2.7	1.767	0.135	0.494	0	0.187	0.873	Lungalunga
Mlali	0.623	0.613	1.71	1.74	1.348	0.199	0.227	0.187	0	0.52	Mikese
Texas	0.992	0.099	0.94	0.94	1.711	0.592	0.876	0.873	0.52	0	Goshuha

*Note:* Values in bold indicate the distance of individuals from Africa assigned to the probable source population. The distance of individuals assigned to the source populations is underlined.

### 3.4 Sequencing, mapping, and assembly of five new complete *D. citri* mitogenomes

The Illumina MiSeq run resulted in an average of 17.6 million reads for each sample, with an average read length of 151 bp. Average GC and AT content were 41% and 59%, respectively. Average Q20 (ratio of bases with phred quality score > 20) was 99%, and average Q30 (ratio of bases with phred quality score > 30) was 95% (see Figure S2). The number of assembled reads ranged between 41, 125 (Tanzania), and 183,235 (China), and sequence average coverage ranged from 414× (Tanzania) to 1845× (China) (see Figure S3).

### 3.5 General mitogenomic features

The five *D. citri* mitogenomes had the typical Metazoan complement of 13 PCGs, 22 transfer tRNAs, two rRNAs, and an AT‐rich noncoding region generally assumed to control transcription and replication. Gene order was identical to that of the *D. citri* mitogenomes publicly available and other species in the family Psyllidae, and identical to the hypothetical ancestral mitogenome organization in insects (Boore, 1999). Twenty‐three genes were located on the majority strand (J‐strand), and the other 14 genes on the minority strand (N‐strand). The complete mitogenome sequences had an average total size of 14,995 bp, similar to that of the sequence (KU647697) used as a reference for mapping and assembly of NGS reads (14,996 bp).

### 3.6 Protein‐coding genes, noncoding AT‐rich region, and intergenic and overlapping regions

The average combined length of the 13 PCGs was 10,798 bp and varied between *ND5* (1618 bp) and ATP8 (153 bp) (see Table S6). The size of the PCGs was similar in the other members of the family Psyllidae, which had an average combined PCG length of 10,814 bp. The large rRNA gene (16s rRNA; 1134 bp) was located between tRNA^Leu1^ and tRNA^Val^, and the small rRNA gene (12s rRNA; 758 bp) was located between tRNA^Val^ and the AT‐rich region. The complete complement of 22 tRNAs was successfully identified with ARWEN software, and tRNA sizes varied between 56 bp (tRNA^Ser1^) and 70 bp (tRNA^Lys^). The typical clover‐leaf structure was predicted for all tRNAs except tRNA^Ser1^, for which the DHU arm was reduced to a simple loop, as commonly observed in metazoans (Bernt, Braband, Schierwater & Stadler, 2013). The five mitogenomes were equally compact, with 10 short gene overlaps (maximum = 11 bp, between tRNA^Glu^ and tRNA^Phe^). Intergenic regions were found at nine locations representing an averaged total of 69 bp, the longest of which was between tRNA^ser^ and ND1 (24 bp). The largest noncoding region (902 bp), located between 12 s rRNA and the tRNA^Ile^‐tRNA^Gln^‐tRNA^Met^ cluster, was annotated as the AT‐rich region.

### 3.7 Nucleotide composition and strand asymmetry

The complete mitogenomes had the high A+T content typical of insects, with values higher than 74.3% in all individual genes (see Table S7). The average A+T content of the AT‐rich region (85.9%) was higher than the average for the complete sequence (74.4%), the combined tRNAs (75.1%), and the two rRNAs (76.9%). Nine PCGs (COII, ATP6, ATP8, COIII, ND3 ND6, CYTB, and ND2) had negative AT skews, and four (ND5, ND4, ND4L, and ND1) had positive AT skews. GC skews were negative for all PCGs.

### 3.8 Mitogenomic comparisons between Africa, China, and the USA

**FIGURE 4:**
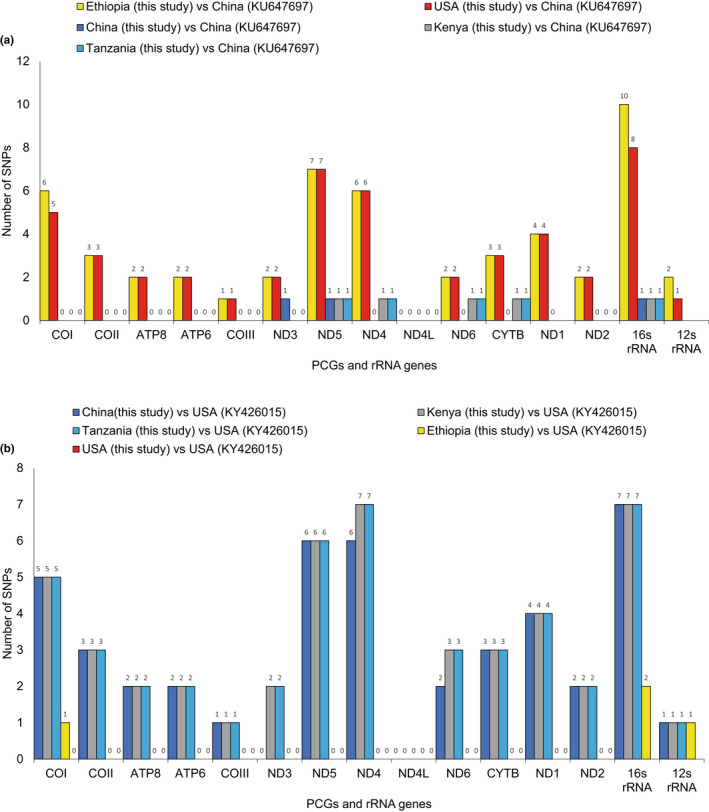
Nucleotide pairwise differences in the mitochondrial protein‐coding and ribosomal RNA genes between five new *Diaphorina citri* mitogenomes (Ethiopia, China, Tanzania, Kenya, and the USA) and (a) a mitogenome from China (GenBank KU647697) and (b) a mitogenome from the USA (GenBank KY426015)

In the PCGs, the highest number of single nucleotide polymorphisms (SNPs) was observed in COI, ND4, and ND5, whereas ND4L was completely conserved. In Africa, Kenya and Tanzania had almost identical mitogenomes, which diverged from Ethiopia by 40 SNPs (nucleotide p‐distances = 0.44%). The mitogenomes from the USA (KY426015) and Kenya/Tanzania differed by an average of 39 SNPs. In contrast, there was only one SNP between the USA (KY426015) and Ethiopia. The mitogenome from China (KU647697) was highly similar to Kenya and Tanzania, with an average of three SNPs. In contrast, China (KU647697) and Ethiopia were separated by 40 SNPs (Figure 4). The sequences from Kenya and Tanzania diverged from the sequence from China by 0.12% and diverged from the sequence from the USA by 0.43%. The sequence from Ethiopia diverged from the sequence from China by 0.43% and diverged from the sequence from the USA by 0.05%. The new sequences from China, Kenya, and Tanzania (hereon referred to as Group 1) and the new sequences from Ethiopia and the USA (Group 2) were separated by an average of 40 SNPs (nucleotide p‐distances = 0.41%) (Figure 4). The total number of substitutions between the two groups comprised 34 transitions and five transversions (see Table S8). Group 1 and Group 2 also differed in the nucleotide sequence in three tRNAs: a U‐C change in the TΨC in tRNA^Phe^, an additional A on the TΨC in tRNA^Asn^ (Group 1), and a U‐C change in the variable loop of tRNA^Ser1^ (see Figure S4).

### 3.9 Phylogeographic structure and genetic diversity of *D. citri*

The phylogeographic structure of *D. citri* in Africa was assessed in the context of COI and 16S rRNA gene regions generated in previous studies from specimens collected in Asia and the Americas using a MJ network and a ML tree.

**FIGURE 5:**
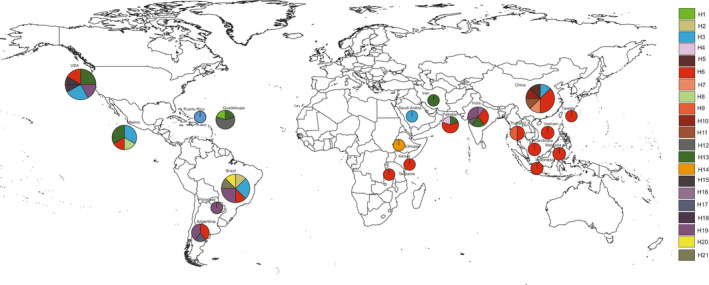
Worldwide geographic distribution of 21 COI haplotypes of *Diaphorina citri*

The MJ network revealed a total of 21 haplotypes distributed among Africa, Asia, and the Americas (Figure 5). Asia had 11 haplotypes with four shared haplotypes, and sequences from the Americas comprised 13 haplotypes with five shared haplotypes. The haplotype with the highest frequency (H6) was found in all world regions except the Middle East (Iran and Saudi Arabia), Ethiopia, Guadeloupe, Paraguay, and Puerto Rico. The sequence from Ethiopia represented a singleton (H14).

**FIGURE 6:**
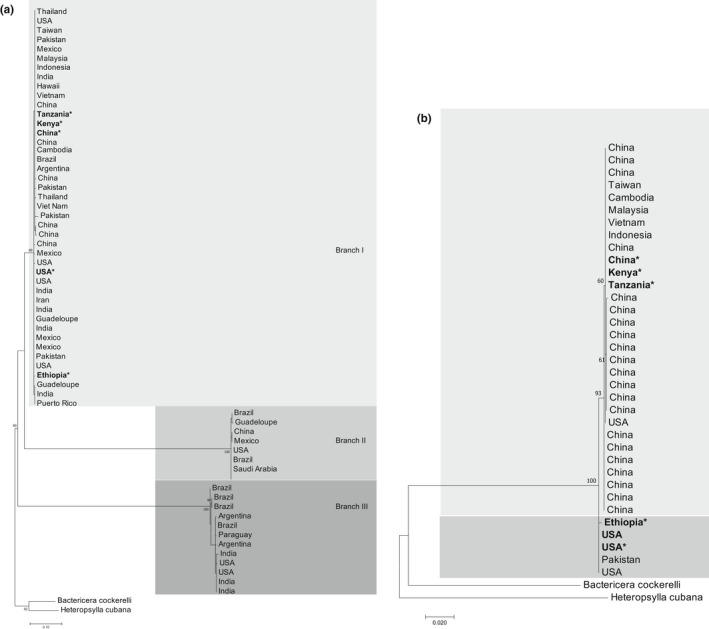
Maximum‐likelihood tree showing the relationships among haplotypes of *Diaphorina citri* from Africa and other world regions using (a) an 874‐bp alignment of 57 COI sequences and (b) using a 1500‐bp alignment of 29 16S ribosomal RNA sequences. *Bactericera cockerelli* and *Heteropsylla cubana* were used as outgroups. Nodal support was calculated using 1000 bootstrap replicates. The length of the branches is proportional to the number of substitutions per site

The COI ML tree recovered three distinct branches (Branch I‐III). The new sequences, represented by 12 closely related haplotypes (H4–H15) seen in the MJ network, formed Branch I along with most sequences from Asia, the Americas, and the Caribbean. Branch II was also formed by sequences from Asia, the Americas, and the Caribbean. Branch III was formed by six haplotypes (H16–H21) exclusively from Asia and the Americas (Figure 6a).

**FIGURE 7:**
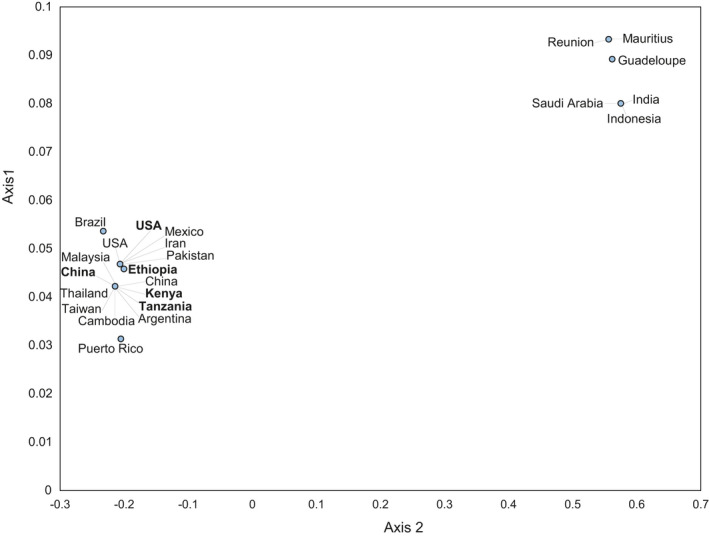
Principal coordinate analysis plot showing genetic distances among 57 *Diaphorina citri* COI sequences based on p‐distances, computed using the classic multidimensional scaling function “cmdscale” in R version 3.5.1

The 16S rRNA ML tree recovered two distinct branches: the new sequences from China, Kenya, and Tanzania fell on the same branch with all sequences from Asia, while Ethiopia, the USA, and Pakistan formed the second branch (Figure 6a). The COI p‐distances evidenced the divergence of the Ethiopian *D. citri* sequence, although this singleton haplotype was less diverged from the sequence from the USA (0.24%) than from the Kenyan, Tanzanian, and Chinese sequences (0.72%) (see Table S9). The principal coordinate analysis (PCoA) plot based on the p‐distances showed an overlap of the new and publicly available *D. citri* from China, Kenya, and Tanzania, and an overlap between the new and publicly available *D. citri* from the USA (Figure 7). The sequence from Ethiopia was positioned closer to the USA cluster than to the other African sequences.

## 4 DISCUSSION

The recent invasion and rapid spread of the Asian citrus psyllid (*D. citri*) in eastern Africa and the confirmed presence of *Candidatus* Liberibacter asiaticus (Las) in Ethiopia and Kenya have raised concerns over the threat of HLB to other citrus‐producing countries in the continent. Our aim was to examine population structure and gene flow within East Africa and to place regional diversity and structure within a global context. For that purpose, we generated data from nuclear DNA markers and complete mitochondrial genomes of an extensive sampling of *D. citri* in East African countries (Ethiopia, Kenya, and Tanzania), China, and the USA. We also provide a depiction of the global phylogeographic structure of the psyllid based on the COI and 16S rRNA sequences.

Across the African locations sampled in this study, Ethiopia was the most diverged as indicated by the low levels of estimated gene flow between Ethiopia and all other sampling sites, and by the Bayesian clustering analysis of microsatellite genotypes. The genotypic assignment of the Ethiopian *D. citri* to populations from the USA and its significant separation from the Kenyan and Tanzanian *D. citri* indicates a separate origin for the Ethiopian population. As the region in Ethiopia where the *D. citri* were collected is characterized by steep mountains, which potentially disrupts natural dispersal of the insect, the psyllid could have been introduced through human‐mediated transportation of rootstock and seedlings infested with eggs and nymphs.

**FIGURE 8:**
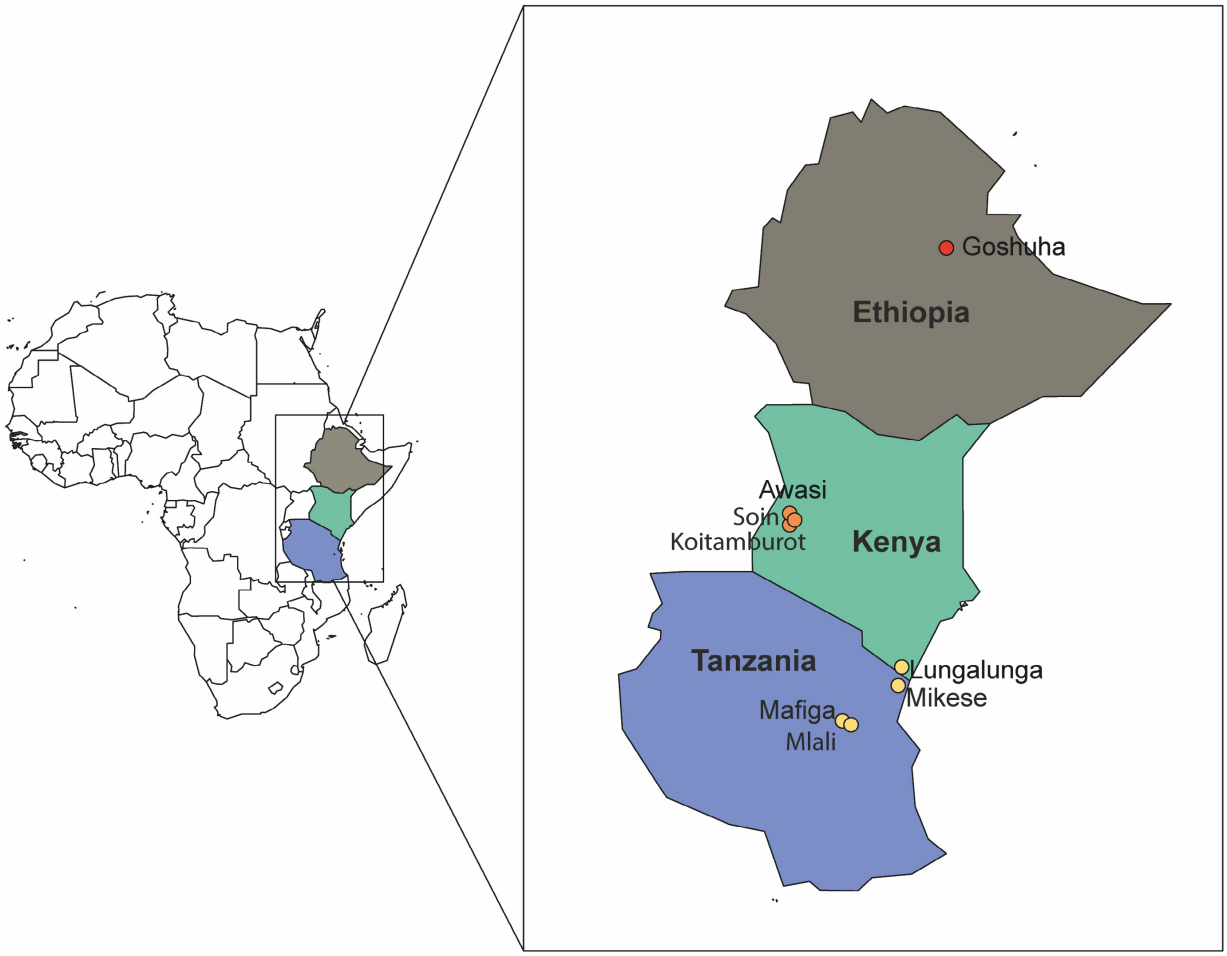
Map of the East African *D. citri* collections. Nodes are colored based on shared genotypes

As for the other eastern African populations, the most probable source of introduction of *D. citri* into Tanzania and Kenya is southeast Asia as the analysis of the minimum spanning tree and the p‐distances showed low divergence between the Chinese and the Tanzanian *D. citri*, as well as the *D. citri* from Lungalunga (Kenya). Low population differentiation between the Kenyan and Tanzanian *D. citri* was expected as there are direct trade routes between the sampled locations, and no significant physical barriers such as impassable mountain ranges or vast deserts to prevent the natural movement of the psyllid. However, western Kenya (Awasi, Soin, and Koitamburot) showed some genetic differentiation from Lungalunga (located in the coastal region) and Tanzania. This pattern may be associated with the geographic proximity of Lungalunga (which lies at the border with Tanzania) to Mikese, Mlali, and Mafiga, as well as the relative distance from the sites in western Kenya, which are farther inland (Figure 8).

Furthermore, there is a direct trade route between the Tanzanian sites and Lungalunga, while there are more physical barriers between Lungalunga and the sites in western Kenya. The *D. citri* populations were largely panmictic; thus, the genetic differentiation among the Kenyan populations may be the result of physical barriers that limit gene flow from Lungalunga towards inland regions resulting in its separation from the populations in western Kenya. Therefore, the movement of the psyllid inland into Kenya is most likely the result of a combination of trade practices and geographical barriers to dispersal. This migration pattern was also seen in other invasive species such as *Bactrocera correcta* (Bezzi) and *Bactrocera dorsalis* (Hendel) in China (Aketarawong et al., 2007; Shi et al., 2012; Qin, et al., 2016) and *Bactrocera invadens* (Drew) in Africa (Khamis et al., 2009).

A recent study based on complete mitochondrial genomes of *D. citri* concluded that the psyllid in California was likely not introduced from China but from somewhere in the southeastern USA (Wu et al., 2017). Based on COI sequences, the recently detected *D. citri* in Ethiopia showed a close phylogenetic relationship with *D. citri* from the Americas (Ajene et al., 2020a).

The comparison of complete *D. citri* mitogenomes from China, Ethiopia, Kenya, Tanzania, and the USA revealed two groups. Group 1 was composed of the sequences from China, Kenya, and Tanzania, and Group 2 included the sequences from Ethiopia and the USA. The mitogenomes of the psyllids from Kenya and Tanzania were highly similar, and different from Ethiopia. Albeit at a low level, *D. citri* from Ethiopia was closer to *D. citri* from the USA than to Kenya or Tanzania. All members of Group 1 had the same number of SNPs when compared to Group 2 and vice versa. This clearly distinguished the two groups in addition to the p‐distances of the complete PCG sequences. Furthermore, the variation in tRNAs between Group 1 and Group 2 showed a similar pattern, with the sequences from Group 1 showing no differences in all tRNAs, while Group 1 and Group 2 had differences in tRNA^Phe^, tRNA^Asn^, and tRNA^Ser1^. Variation in the tRNA^Asn^ was also reported between mitogenomes from the USA and China (Wu et al., 2017). Therefore, our results agree with the previous report of two genetic clusters associated with the USA and China, identified by mitogenome analyses (Wu et al., 2017).

The COI‐based ML tree showed three main branches, and all sequences generated in this study clustered on Branch I, which also included *D. citri* from Asia and America. The COI‐based MJ network clustered the samples from Kenya and Tanzania in the same haplotype with China and Vietnam, while the Ethiopian sequence had a unique haplotype. This singleton haplotype was also found when *D. citri* was first detected in Ethiopia (Ajene et al., 2020a). Furthermore, COI‐based genetic divergence among African psyllids showed no difference between the Kenyan and Tanzanian sequences but revealed divergence between these and Ethiopia (p‐distance = 0.72%). In comparison with the probable source populations (China and the USA), Kenyan and Tanzanian *D. citri* were identical to the Chinese while the Ethiopian *D. citri* were more similar to those from the USA than to those from China.

The 16S rRNA could be a viable option for differentiating *D. citri* populations, as has been shown in *B. cockerelli* (Swisher & Crosslin, 2014). Furthermore, the high polymorphism in ND5 and CytB as seen in our study and reported by Wu et al. (2017) makes them suitable candidates for assessing intraspecific insect differentiation. In our study, 16S rRNA was the gene that had the highest number of SNPs across the complete mitogenomes. The ML tree using 16S rRNA sequences further showed the differentiation between samples from Africa, America, and Asia: Ethiopia clustered with the USA, while Kenya and Tanzania clustered with China and other southwestern Asian samples. A multi‐gene approach to intraspecies differentiation is often needed to provide a clearer picture of the phylogeographic structure of insect species. For example, COI together with ND5 has been used for phylogenetic studies of flies (Navajas et al., 1996), CytB has been shown to be better than COI at recovering population structure in *Athetis lepigone* (Chen et al., 2019), COIII has been used in intraspecific studies of *Ixodes pacificus* (Kain et al., 1999), and comparisons of complete mitogenomes showed that the most polymorphic regions of two Saturniidae species were located in ATP6, ND4, ND5, ND6, and CYTB (Langley et al., 2020). However, previous studies on the phylogeography of American and Asian *D. citri* have relied only on analyses of COI sequences (De León et al., 2011, Boykin et al., 2012; Guidolin & Consoli, 2013; Lashkari et al., 2014). Boykin et al. (2012) identified eight haplotypes from 16 countries in five regions and concluded that *D. citri* from North America is more closely related to southwestern Asia, whereas *D. citri* from South America is closely related to *D. citri* from southwestern and southeastern Asia. Similarly, our results showed that the Asian psyllids that shared haplotypes (H3, H12, H13, and H19) with the Americas were the southwest (India and Pakistan) and Middle East (Iran and Saudi Arabia) populations. Therefore, *D. citri* from the Americas are more closely linked to *D. citri* from southwestern Asia and the Middle East with four of five shared haplotypes only containing psyllids from these two regions. In addition, the singleton from Ethiopia was more closely linked to the American and southwestern Asia/Middle Eastern *D. citri*. Therefore, *D. citri* in Africa seems to have been introduced in separate events. While *D. citri* was likely introduced in Tanzania and Kenya through the shipping routes from China with infested plant material (Rwomushana et al., 2017), the probable route of introduction of *D. citri* into Ethiopia is still unknown. Furthermore, the timeline of *D. citri* detections, as well as the locations (coastal and border regions of Kenya and Tanzania) of detections, seems to suggest a scenario of movement of the psyllid between Tanzania and Kenya, while *D. citri* in Ethiopia was first detected 3 years later in the north‐eastern part of the country (Ajene et al., 2020a).

Nuclear microsatellite markers, which are biparentally inherited, makes them more suitable for gene flow analyses than mitochondrial DNA. Our microsatellite‐based analyses showed two distinct populations of *D. citri* in Africa with likely different origins. We also estimated relatively low gene flow among most Kenyan and Tanzanian populations, with the exception of Lungalunga. Overall, these results indicate that the first introduction of *D. citri* into East Africa occurred in Tanzania with psyllids from southeastern Asia, followed by a dispersal inland, and the psyllids found in Ethiopia most likely originated from the Americas.

This hypothesis is also supported by the analyses of COI and 16S rRNA sequences: The Kenyan and Tanzanian *D. citri* grouped with the southeast Asian *D. citri*, while the Ethiopian grouped with the *D. citri* from the Americas, also indicating that the psyllids present in the three African countries were not introduced from the same source. The combined evidence from the complete mitochondrial genomes, and COI and 16S rRNA sequences, showed a distinct phylogeographic structure of *D. citri* in Ethiopia compared with that in Kenya and Tanzania.

In conclusion, our study shows that the most probable introduction of *D. citri* into Kenya and Tanzania occurred from Asia, in accordance with the link between the Chinese *D. citri* population and the populations from Kenya and Tanzania based on the microbiome and endosymbiont composition of the psyllid (Ajene et al., 2020c). We also show that the *D. citri* from Ethiopia represented a distinct introduction event possibly originating from the Americas.

The clarification of the separate introduction routes of *D. citri* and Las in Africa may have implications on HLB regulatory strategies, in particular those aimed at preventing the establishment of Las. This information is of phytosanitary and quarantine regulatory importance for the management of HLB spread in the continent. Accurate determination of the origin of *D. citri* populations in Africa and its population structure and diversity across a wider geographic range on the continent will provide further insights and hopefully strengthen the argument for stricter quarantine and regulatory policies.

## AUTHOR CONTRIBUTIONS

**Inusa Jacob Ajene:** Conceptualization (supporting); data curation (equal); formal analysis (lead); investigation (lead); methodology (equal); software (lead); validation (equal); visualization (equal); writing – original draft (equal); writing – review and editing (equal). **Fathiya Khamis:** Conceptualization (equal); supervision (equal); writing – original draft (equal); writing – review and editing (equal). **Barbara van Asch:** Conceptualization (equal); formal analysis (supporting); supervision (equal); writing – original draft (equal); writing – review and editing (equal). **Gerhard Pietersen:** Supervision (equal); writing – original draft (equal); writing – review and editing (equal). **Nurhussein Seid:** Data curation (equal). **Anne Wairimu Wambui:** Formal analysis (supporting); investigation (supporting). **Fidelis Levi Ombura:** Data curation (equal). **Komivi Senyo Akutse:** Data curation (equal). **Mamoudou Sétamou:** Data curation (equal); writing – review and editing (equal). **Sevgan Subramanian:** Resources (equal). **Samira Mohamed:** Conceptualization (equal); funding acquisition (equal); project administration (equal); resources (equal). **Sunday Ekesi:** Conceptualization (equal); funding acquisition (equal); project administration (equal); resources (equal); supervision (equal); writing – review and editing (equal).

## CONFLICT OF INTEREST

The authors declare no conflict of interest.

## Supporting information


Appendix S1

## Data Availability

The mitogenome sequence data that support the findings of this study are openly available in GenBank at https://submit.ncbi.nlm.nih.gov/subs/bioproject/SUB10405082/overview, GenBank BioProject PRJNA764842. All other data that support the findings of this study are available from the corresponding author upon reasonable request
