# Supplementary material for: Genetic diversity of Diaphorina citri (Hemiptera: Liviidae) unravels phylogeographic structure and invasion history of eastern African populations
Source: Ecol Evol. 2022 Jul 17;12(7):e9090. doi: 10.1002/ece3.9090 (PMC9289372; doi:10.1002/ece3.9090)
Supplement: Supplementary file 1 — Appendix S1 [file ECE3-12-e9090-s001.docx]

**Supplemental Information for:**

**Genetic diversity of *Diaphorina citri* (Hemiptera: Liviidae) unravels phylogeographic structure and invasion history of Eastern African populations**

Inusa Jacob Ajene^1,2,3^, Fathiya Mubarak Khamis^2^*, Barbara van Asch^3^, Gerhard Pietersen^3^, Nurhussen Seid^4^, Anne Wambui Wairimu^2^, Fidelis Levi Ombura^2^, Komivi Senyo Akutse^2^, Mamoudou Sétamou^5^, Sevgan Subramanian^2^, Samira Mohammed^2^ and Sunday Ekesi^2^

^1^Department of Crop Protection, Faculty of Agriculture Ahmadu Bello University, Zaria, Nigeria, ^2^International Center of Insect Physiology and Ecology, Nairobi, Kenya, ^3^Department of Genetics, Stellenbosch University, Stellenbosch, South Africa, ^4^Ethiopian Institute of Agricultural Research, Addis Ababa, Ethiopia, ^5^Texas A&M University, Kingsville Citrus Centre, Weslaco, Texas, USA.

**
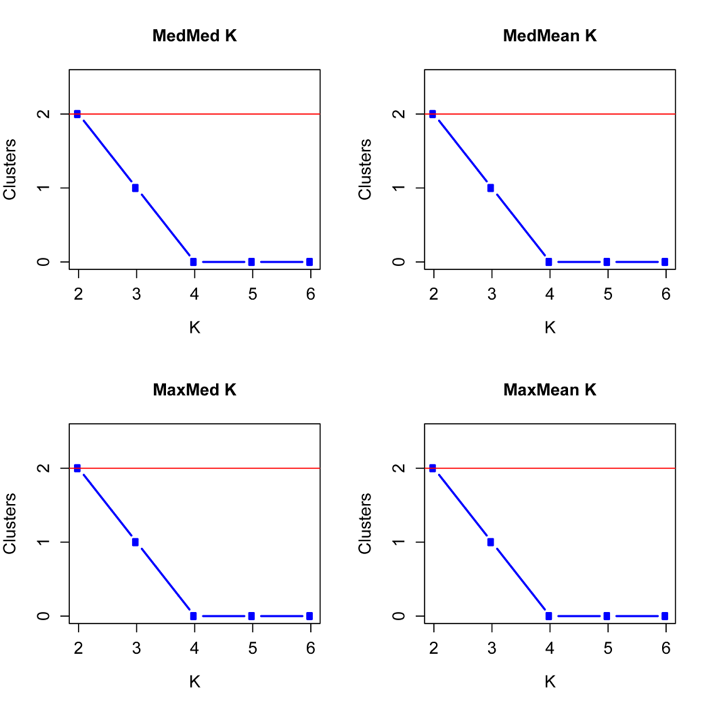
**

**Fig. S1.** Bayesian analysis of 270 *Diaphorina citri* individuals based on 10 microsatellite loci showing optimal number of clusters as inferred by Med- and Mean K values. The optimal K (Y-axis) used to explain predefined 10 populations after removing spurious clusters are indicated by red lines.

**
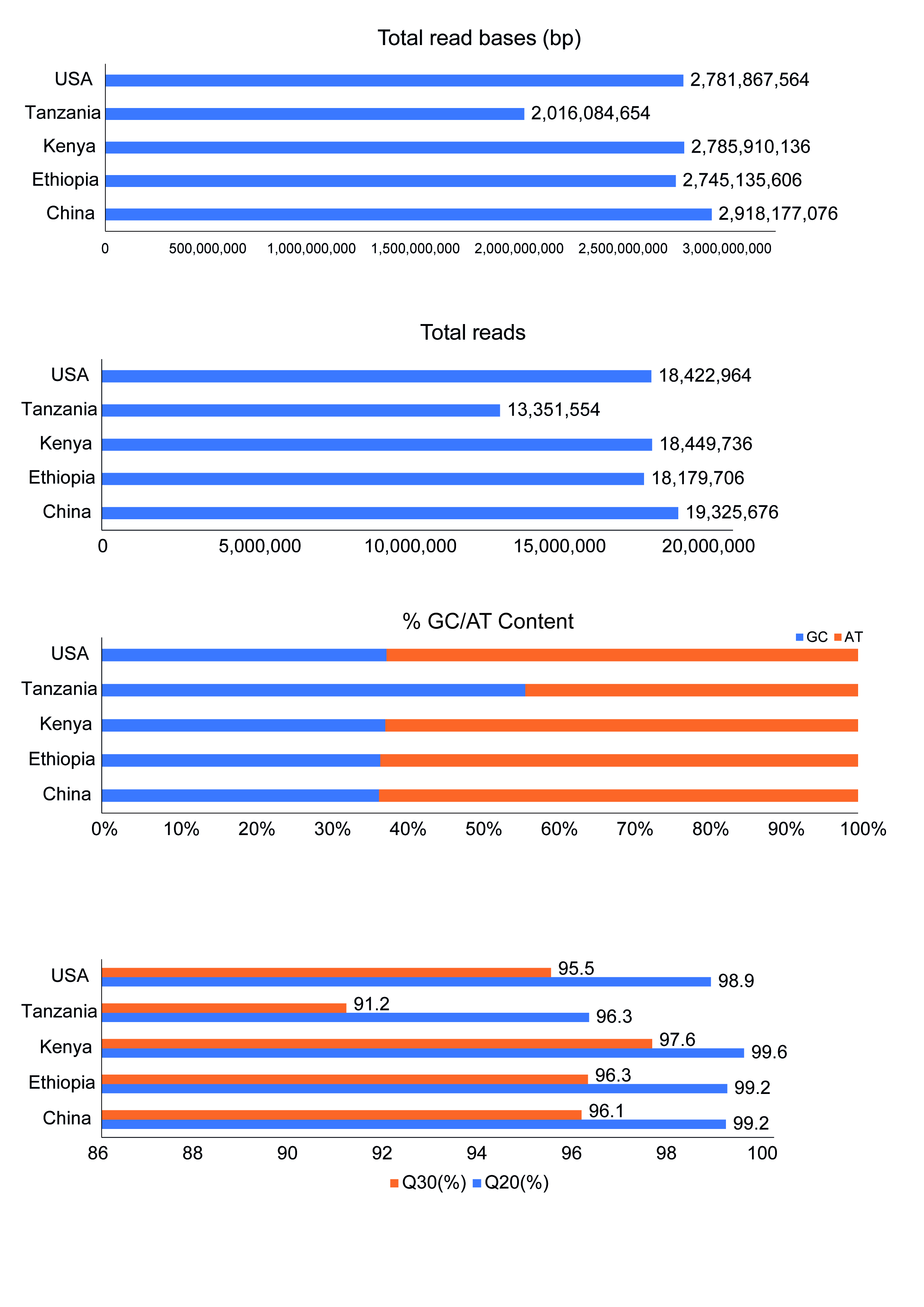
**

**Fig. S2**. Summary statistics of Illumina MiSeq sequencing of five individual *Diaphorina citri* specimens from China, Ethiopia, Kenya, Tanzania and USA. (a) Total number of base pairs for each sample. (b) Total number of reads. (c) Percentage of GC/AT content. (d) Percentage Q20/Q30.

**
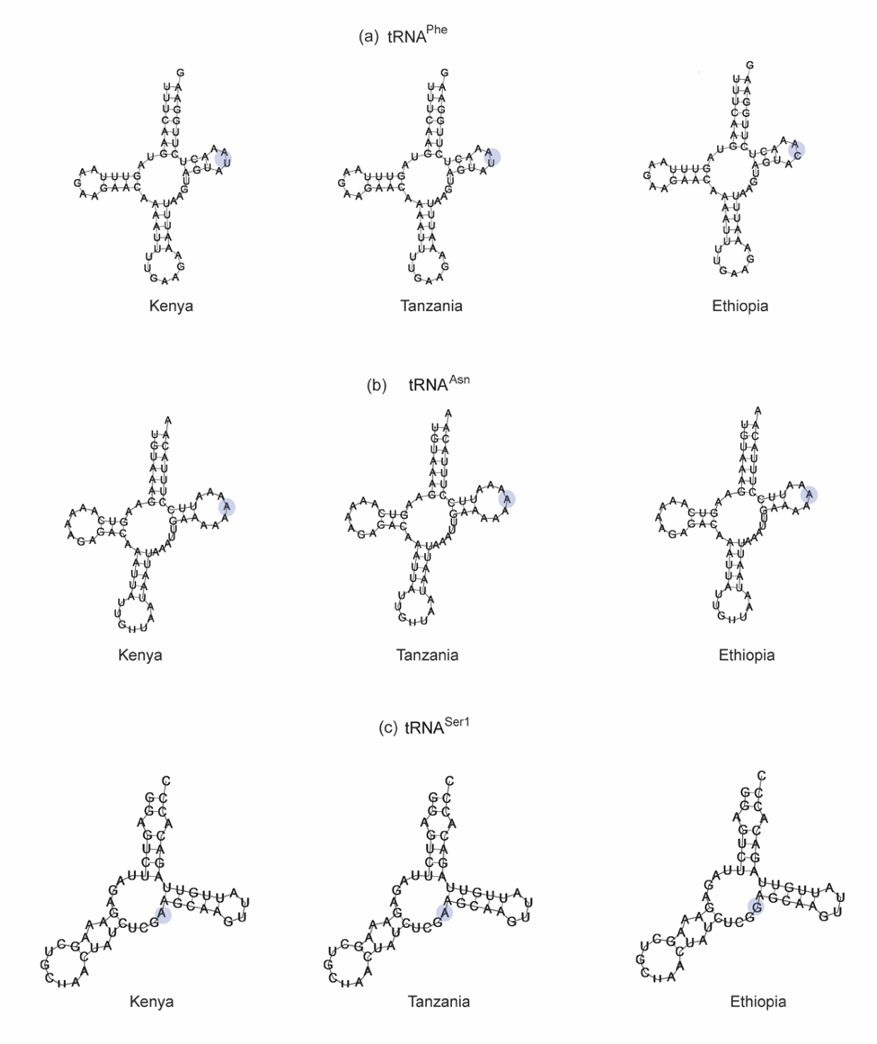
**

**Fig. S3.** Schematic representation of the sequencing coverage of five new mitochondrial genomes of *Diaphorina citri* from China, Ethiopia, Kenya, Tanzania and USA. Reference-based mapping and assembly of the reads was performed using a publicly available *D. citri* sequence (KU647697).

**
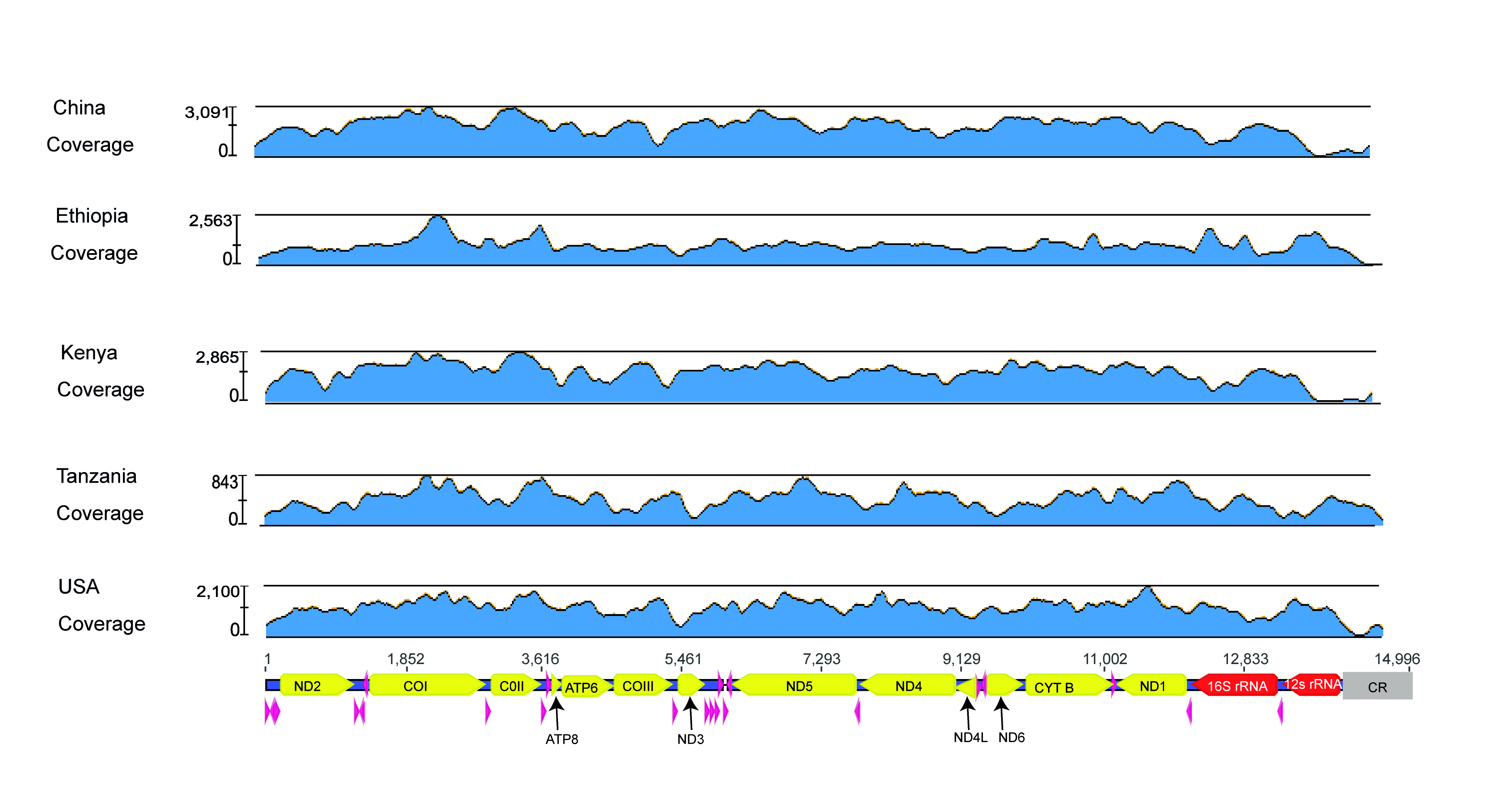
**

**Fig. S4.** Variation in the sequences of three tRNA genes (a) *tRNA^Phe^,*(b) *tRNA^Asn^* and (c) *tRNA^Ser1^*, in the mitochondrial genomes of *Diaphorina citri* from Ethiopia, Kenya and Tanzania. Bases highlighted in blue indicate differences in the sequences.

**Table S1.** Microsatellite markers used in PCR amplification and genotyping of the simple sequence repeats in *Diaphorina citri* in this study. Ta – Annealing temperature.

| **Locus** | **Primer sequence** | **Repeat sequence** | **Size range (bp)** | **Ta (°C)** | **Flourescent dye** | **Multiplex** |
| --- | --- | --- | --- | --- | --- | --- |
| Dci01 | F: TTTCGAAGACCCAAACAACC | (TTTA)3(TTA)6 | 197–222 | 63.7 | VIC | Dci05,Dci04 |
|  | R: TCCTGCCTTCCATCTCTCTT |  |  |  |  |  |
| Dci02 | F: GGTGAACGAAAACAAAGGAGA | (CTATT)4 | 190–200 | 53.5 | NED | Dci03,Dci07 |
|  | R: CGGGGTGATAGGTCTCTAGC |  |  |  |  |  |
| Dci03 | F: GAAGGATGCCAAGAAAGCAC | (GCT)8N21(GCT)4 | 209–227 | 55.1 | VIC | Dci02,Dci07 |
|  | R: TCGGCACATTCTTCTTCACA |  |  |  |  |  |
| Dci04 | F: CCAGCGTGCTAAAACTCAAA | (CTT)4 | 294–304 | 57.4 | NED | Dci01,Dci05 |
|  | R: TTGATGCAAAAAGGAACAAAAA |  |  |  |  |  |
| Dci05 | F: CCCCCAAGAGACAAGTTCAA | (TGA)5 | 330–340 | 52.1 | FAM | Dci01,Dci04 |
|  | R: TCCTTGTTCAACGACCATGA |  |  |  |  |  |
| Dci07 | F: CGGCAGTCCCAGTAGGTAAG | (TAGG)3 | 263–275 | 54 | FAM | Dci02,Dci03 |
|  | R: GAATTCGTCGCTTCCCAATA |  |  |  |  |  |
| Dci09 | F: CATCCAAAGGAGCGACACTT | (GAA)24 | 174–186 | 52.9 | VIC | Dci10 |
|  | R: TCCTTTTCCCCTTCTCCTGT |  |  |  |  |  |
| Dci10 | F: GAAGAAAGAGGGGAAGAGG | (GAA)26 | 210–262 | 48 | PET | Dci09 |
|  | R: CGACTTCACCAGGAGAGAAAG |  |  |  |  |  |
| Dci11 | F: GGTCTGCCAACTTGTCCATT | (GA)10(GGGA)2(GA)4 | 242–260 | 62.7 | FAM | Dci12 |
|  | R: CCCCCTCTTACCTCGTCTCT |  |  |  |  |  |
| Dci12 | F: GGTGAGTGCTCTCCTTTCCA | (CT)4 | 211–223 | 56.7 | NED | Dci11 |
|  | R: CTGCAGACATCCTGCATACAA |  |  |  |  |  |

**Table S2.** List of publicly available *Diaphorina citri* mitogenome sequences (n = 31) used for the estimation of pairwise genetic divergence.

| **Country** | **GenBank accession** | **Reference** |
| --- | --- | --- |
| Cambodia | MF614824 | Wu et al., 2017 |
| China | NC_030214 | Wu et al., 2016 |
|  | MG489916 | Wu et al., 2017 |
|  | MF614803 -MF614822 | Wu et al., 2017 |
|  | MF426268 | Percy et al., 2017 |
|  | KX073968 | Xiong et al., 2016 |
|  | KU647697 | Wu et al., 2016 |
| Indonesia | MF614827 | Wu et al., 2017 |
| Malaysia | MF614825 | Wu et al., 2017 |
| Pakistan | MF614828 | Wu et al., 2017 |
| Taiwan | MF614823 | Wu et al., 2017 |
| USA | KY426015 | Wu et al., 2017 |
|  | KY426014 | Wu et al., 2017 |
| Vietnam | MF614826 | Wu et al., 2017 |

**Table S3.** List of publicly available and new COI sequences (n = 573) *Diaphorina citri* used for the construction of a median-joining network, and a maximum likelihood tree.

| **Country** | **GenBank accession** | **Reference** |
| --- | --- | --- |
| Argentina | KY001577 | Chu et al., 2019 |
| Brazil | KC011223 - KC011243 | Guidolin & Consoli 2013 |
|  | KC354739 - KC354785 | Guidolin & Consoli 2013 |
|  | FJ190228 - FJ190333 | Boykin et al., 2012 |
| Cambodia | KU941172 - KU941176 | Fuentes et al., 2018 |
| China | MH970587- MH970829 | Zhang & Xia 2018 |
|  | MF140371 - MF140373 | Qasim et al., 2019 |
|  | KU940421 - KU941158 | Fuentes et al., 2018 |
|  | MH001384 | Fuentes et al., 2018 |
|  | FJ190357 - FJ190368 | Boykin et al., 2012 |
|  | MK804829 - MK804888 | Luo & Fu 2019 |
|  | FJ190297 -FJ190299 | Boykin et al., 2012 |
| Guadeloupe | FJ190346 - FJ190356 | Boykin et al., 2012 |
| India | FJ190342 - FJ190345 | Boykin et al., 2012 |
|  | KU050675 | Das et al., 2009 |
|  | KR865959 - KR865960 | Das et al., 2009 |
| Indonesia | KU941145 - KU941149 | Fuentes et al., 2018 |
|  | FJ190264 - FJ190336 | Boykin et al., 2012 |
| Iran | KC509563 - KC509572 | Lashkari et al., 2013 |
| Malaysia | KU941150 - KU941154 | Fuentes et al., 2018 |
| Mauritius | FJ190312 - FJ190316 | Boykin et al., 2012 |
| Mexico | MH001383 | Fuentes et al., 2018 |
|  | FJ190300 - FJ190305 | Boykin et al., 2012 |
|  | KJ453889 - KJ453897 | Sanchez et al., 2014 |
|  | FJ190306 - FJ190309 | Boykin et al., 2012 |
| Pakistan | FJ190288 - FJ190292 | Boykin et al., 2012 |
|  | MH001373 - MH001379 | Fuentes et al., 2018 |
|  | KY001579 | Chu et al., 2019 |
|  | KC509561 - KC509562 | Lashkari et al., 2013 |
|  | MF140365 - MF140370 | Qasim et al., 2019 |
|  | KU941179 - KU941184 | Fuentes et al., 2018 |
| Puerto Rico | FJ190260 - FJ190262 | Boykin et al., 2012 |
| Reunion | FJ190317 - FJ190319 | Boykin et al., 2012 |
| Saudi Arabia | FJ190337 - FJ190341 | Boykin et al., 2012 |
| Taiwan | FJ190283 - FJ190287 | Boykin et al., 2012 |
| Thailand | MH001380 | Fuentes et al., 2018 |
|  | KY001580 | Chu et al., 2019 |
|  | KU941177 - KU941178 | Fuentes et al., 2018 |
|  | FJ190293 - FJ190296 | Boykin et al., 2012 |
| USA | MH001381 - MH001382 | Fuentes et al., 2018 |
|  | KY001578 | Chu et al., 2019 |
|  | KU941185 - KU941204 | Fuentes et al., 2018 |
|  | FJ190187-FJ190241 | Boykin et al., 2012 |
| Vietnam | FJ190273 - FJ190378 | Boykin et al., 2012 |
|  | KU941155 - KU941162 | Chu et al., 2019 |

**Table S4.** Genetic variability estimates in samples of *Diaphorina citri* from five countries. N: number of alleles; Na: mean number of alleles; Ne: mean number of effective alleles; HO: mean observed heterozygosity; HE: mean expected heterozygosity; uHe: unbiased expected heterozygosity; F: fixation index

| **Country** | **Population** | **N** | **Na** | **Ne** | **Ho** | **He** | **uHe** | **F** |
| --- | --- | --- | --- | --- | --- | --- | --- | --- |
| Kenya | Awasi | 2.1 | 1.7 | 1.401 | 0.500 | 0.300 | 0.431 | -0.715 |
| China | Fuzhou | 1.8 | 1.4 | 1.173 | 0.475 | 0.277 | 0.396 | -0.735 |
| Ethiopia | Goshuha | 3.9 | 3.1 | 2.535 | 0.680 | 0.459 | 0.551 | -0.547 |
| Kenya | Koitamburot | 2.0 | 2.3 | 1.734 | 0.483 | 0.328 | 0.448 | -0.547 |
| Kenya | Lungalunga | 4.4 | 3.3 | 2.531 | 0.700 | 0.473 | 0.565 | -0.525 |
| Tanzania | Mafiga | 3.0 | 2.7 | 2.473 | 0.700 | 0.439 | 0.584 | -0.688 |
| Tanzania | Mikese | 4.6 | 0.8 | 0.680 | 0.300 | 0.164 | 0.17 | -0.852 |
| Tanzania | Mlali | 3.0 | 2.4 | 2.230 | 0.800 | 0.470 | 0.643 | -0.753 |
| Kenya | Soin | 2.4 | 2.3 | 2.000 | 0.700 | 0.410 | 0.604 | -0.787 |
| USA | Texas | 5.8 | 2.3 | 1.802 | 0.583 | 0.380 | 0.447 | -0.581 |

**Table S5.** Average coefficient of ancestry obtained from a Structure run with K = 2 for the 270 individuals of *D. citri* from five countries. Co-ancestry higher than 10% of each population in a cluster is shown in bold.

|  | **Inferred clusters (K)** | | |
| --- | --- | --- | --- |
| **Country** | **Population** | **1** | **2** |
| China | Fuzhou | **0.847** | 0.153 |
| Ethiopia | Goshuha | **0.730** | 0.270 |
| Kenya | Awasi | 0.316 | **0.684** |
|  | Koitamburot | 0.387 | **0.613** |
|  | Lungalunga | 0.338 | **0.662** |
|  | Soin | 0.326 | **0.674** |
| Tanzania | Mafiga | 0.416 | **0.584** |
|  | Mikese | 0.292 | **0.708** |
|  | Mlali | 0.520 | **0.480** |
| USA | Texas | **0.863** | 0.137 |

**Table S6.** Gene order and composition of the complete mitochondrial genomes of *Diaphorina citri* collected in China, Ethiopia, Kenya, Tanzania and the USA. N – majority strand; J – minority strand; IGN – number of intergenic nucleotides (negative values indicate overlapping between genes).


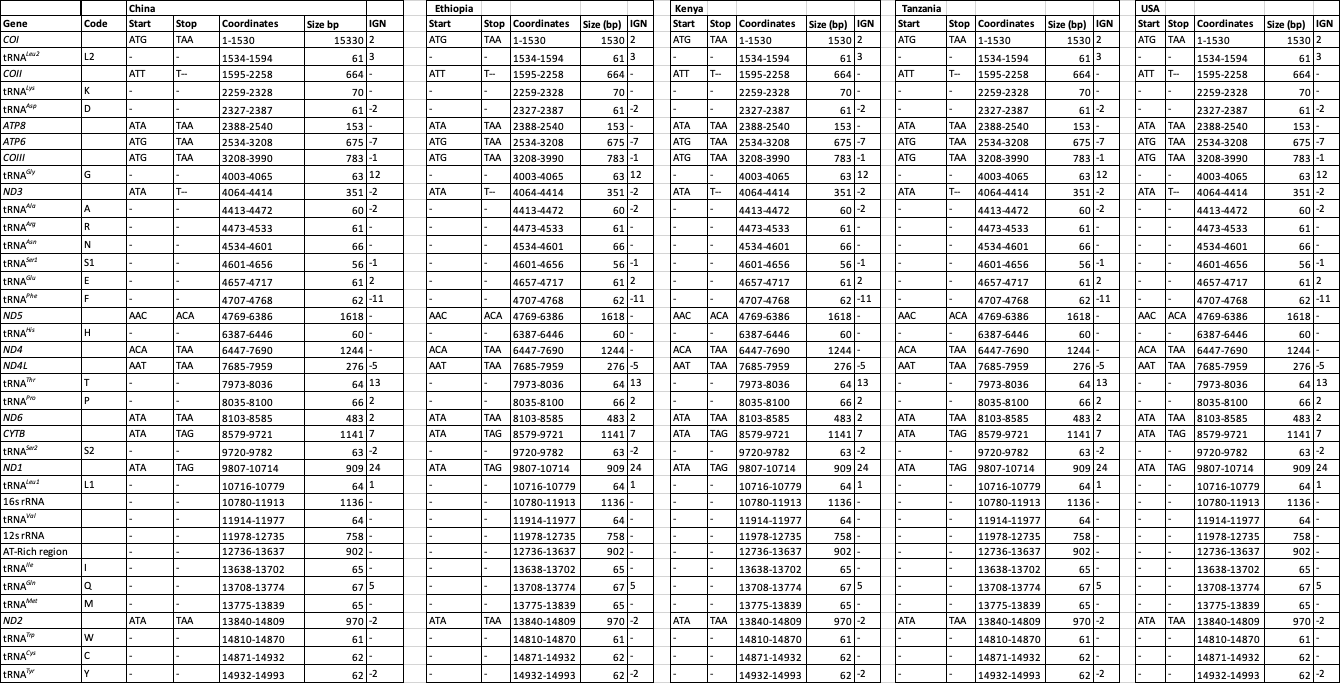


**Table S7.** Nucleotide composition of the complete mitochondrial sequences of five *Diaphorina citri* specimens collected in China, Ethiopia, Kenya, Tanzania and USA. AT-skew = (A - T)/(A + T); CG-skew = (G - C)/(G + C).

| **China** | | | | | | | | | | |
| --- | --- | --- | --- | --- | --- | --- | --- | --- | --- | --- |
| **Region** | **A%** | **C%** | **G%** | **T%** | **A+T%** | **G+C%** | **AT-skew** | **GC-skew** | **Size (bp)** | **Size (%)** |
| *COI* | 32.4 | 17.7 | 13.2 | 36.7 | 69.1 | 30.9 | -0.1 | -0.1 | 1,530 | 10.2 |
| *COII* | 34.8 | 17.6 | 10.4 | 37.2 | 72.0 | 28.0 | 0.0 | -0.3 | 664 | 4.4 |
| *ATP8* | 35.9 | 20.9 | 4.6 | 38.6 | 74.5 | 25.5 | 0.0 | -0.6 | 153 | 1.0 |
| *ATP6* | 36.6 | 17.0 | 8.7 | 37.6 | 74.2 | 25.7 | 0.0 | -0.3 | 675 | 4.5 |
| *COIII* | 36.5 | 15.7 | 10.7 | 37 | 73.5 | 26.4 | 0.0 | -0.2 | 783 | 5.2 |
| *ND3* | 37.8 | 13.5 | 8.3 | 40.4 | 78.2 | 21.8 | 0.0 | -0.2 | 349 | 2.3 |
| *ND5* | 43.9 | 17.0 | 10.2 | 28.9 | 72.8 | 27.2 | 0.2 | -0.3 | 1,618 | 10.8 |
| *ND4* | 46.3 | 18.0 | 8.6 | 27.1 | 73.4 | 26.6 | 0.3 | -0.4 | 1,244 | 8.3 |
| *ND4L* | 50.4 | 14.9 | 5.4 | 29.3 | 79.7 | 20.3 | 0.3 | -0.5 | 276 | 1.8 |
| *ND6* | 33.3 | 16.6 | 6.0 | 44.1 | 77.4 | 22.6 | -0.1 | -0.5 | 483 | 3.2 |
| *CYTB* | 31.9 | 18.2 | 11.0 | 38.8 | 70.7 | 29.2 | -0.1 | -0.2 | 1,141 | 7.6 |
| *ND1* | 46.4 | 16.8 | 11.1 | 25.8 | 72.2 | 27.9 | 0.3 | -0.2 | 912 | 6.1 |
| 16s rRNA | 41.5 | 16.0 | 6.6 | 35.9 | 77.4 | 22.6 | 0.1 | -0.4 | 1,134 | 7.6 |
| 12s rRNA | 40.1 | 16.1 | 8.0 | 35.8 | 75.9 | 24.1 | 0.1 | -0.3 | 758 | 5.1 |
| *ND2* | 36.0 | 16.3 | 8.0 | 39.7 | 75.7 | 24.3 | 0.0 | -0.3 | 970 | 6.5 |
| **PCGs** | **38.6** | **17.1** | **9.9** | **34.4** | **73.0** | **27.0** | **0.1** | **-0.3** | **10,798** | **72.1** |
| tRNAs | 39.8 | 14.8 | 10.2 | 35.3 | 75.1 | 25.0 | 0.1 | -0.2 | 1386 | 9.3 |
| rRNAs | 41.0 | 16.0 | 7.2 | 35.8 | 76.8 | 23.2 | 0.1 | -0.4 | 1892 | 12.6 |
| AT-rich region | 38.9 | 9.0 | 5.0 | 47.1 | 86.0 | 14.0 | -0.1 | -0.3 | 902 | 6.0 |
| **Complete mtDNA** | **39.0** | **16.3** | **9.3** | **35.4** | **74.4** | **25.6** | **0.0** | **-0.3** | **14978** | **100.0** |
|  |  |  |  |  |  |  |  |  |  |  |
| **Ethiopia** | | | | | | | | | | |
| **Region** | **A%** | **C%** | **G%** | **T%** | **A+T%** | **G+C%** | **AT-skew** | **GC-skew** | **Size (bp)** | **Size (%)** |
| *COI* | 32.6 | 17.6 | 13.1 | 36.7 | 69.3 | 30.7 | -0.1 | -0.1 | 1,530 | 10.2 |
| *COII* | 34.6 | 17.3 | 10.4 | 37.7 | 72.3 | 27.7 | 0.0 | -0.2 | 664 | 4.4 |
| *ATP8* | 35.9 | 20.3 | 5.2 | 38.6 | 74.5 | 25.5 | 0.0 | -0.6 | 153 | 1.0 |
| *ATP6* | 36.9 | 17.0 | 8.4 | 37.6 | 74.5 | 25.4 | 0.0 | -0.3 | 675 | 4.5 |
| *COIII* | 36.4 | 15.7 | 10.9 | 37.0 | 73.4 | 26.6 | 0.0 | -0.2 | 783 | 5.2 |
| *ND3* | 38.1 | 13.5 | 8.0 | 40.4 | 78.5 | 21.5 | 0.0 | -0.3 | 349 | 2.3 |
| *ND5* | 44.0 | 16.9 | 10.1 | 28.9 | 72.9 | 27.1 | 0.2 | -0.3 | 1,618 | 10.8 |
| *ND4* | 46.1 | 18.1 | 8.8 | 27.0 | 73.1 | 26.9 | 0.3 | -0.3 | 1,244 | 8.3 |
| *ND4L* | 50.4 | 14.9 | 5.4 | 29.3 | 79.7 | 20.3 | 0.3 | -0.5 | 276 | 1.8 |
| *ND6* | 33.3 | 16.6 | 6.0 | 44.1 | 77.4 | 22.6 | -0.1 | -0.5 | 483 | 3.2 |
| *CYTB* | 31.8 | 18.3 | 11.0 | 38.8 | 70.6 | 29.4 | -0.1 | -0.2 | 1,141 | 7.6 |
| *ND1* | 46.4 | 16.7 | 11.2 | 25.8 | 72.2 | 27.9 | 0.3 | -0.2 | 912 | 6.1 |
| 16s rRNA | 41.8 | 15.8 | 6.4 | 36.0 | 77.8 | 22.3 | 0.1 | -0.4 | 1,134 | 7.6 |
| 12s rRNA | 40.1 | 16.1 | 8.0 | 35.8 | 75.9 | 24.1 | 0.1 | -0.3 | 758 | 5.1 |
| *ND2* | 35.9 | 16.2 | 8.1 | 39.8 | 75.7 | 24.3 | -0.1 | -0.3 | 970 | 6.5 |
| **PCGs** | **38.6** | **17.0** | **9.9** | **34.4** | **73.0** | **26.9** | **0.1** | **-0.3** | **10,798** | **72.1** |
| tRNAs | 39.8 | 14.5 | 10.1 | 35.6 | 75.4 | 24.6 | 0.1 | -0.2 | 1386 | 9.3 |
| rRNAs | 41.1 | 15.9 | 7.1 | 35.9 | 77.0 | 23.0 | 0.1 | -0.4 | 1892 | 12.6 |
| AT-rich region | 39.4 | 9.1 | 4.8 | 46.8 | 86.2 | 23.0 | -0.1 | -0.3 | 902 | 6.0 |
| **Complete mtDNA** | **39.0** | **16.3** | **9.3** | **35.3** | **74.3** | **25.6** | **0.0** | **-0.3** | **14978** | **100.0** |
|  |  |  |  |  |  |  |  |  |  |  |
| **Kenya** | | | | | | | | | | |
| **Region** | **A%** | **C%** | **G%** | **T%** | **A+T%** | **G+C%** | **AT-skew** | **GC-skew** | **Size (bp)** | **Size (%)** |
| *COI* | 32.4 | 17.7 | 13.2 | 36.7 | 69.1 | 30.9 | -0.1 | -0.1 | 1,530 | 10.2 |
| *COII* | 34.8 | 17.6 | 10.4 | 37.2 | 72.0 | 28.0 | 0.0 | -0.3 | 664 | 4.4 |
| *ATP8* | 35.9 | 20.9 | 4.6 | 38.6 | 74.5 | 25.5 | 0.0 | -0.6 | 153 | 1.0 |
| *ATP6* | 36.6 | 17.0 | 8.7 | 37.6 | 74.2 | 25.8 | 0.0 | -0.3 | 675 | 4.5 |
| *COIII* | 36.5 | 15.7 | 10.7 | 37.0 | 73.5 | 26.4 | 0.0 | -0.2 | 783 | 5.2 |
| *ND3* | 38.1 | 13.5 | 8.0 | 40.4 | 78.5 | 21.5 | 0.0 | -0.3 | 349 | 2.3 |
| *ND5* | 43.9 | 17.0 | 10.2 | 28.9 | 72.8 | 27.2 | 0.2 | -0.3 | 1,618 | 10.8 |
| *ND4* | 46.2 | 18.0 | 8.7 | 27.1 | 73.3 | 26.7 | 0.3 | -0.3 | 1,244 | 8.3 |
| *ND4L* | 50.4 | 14.9 | 5.4 | 29.3 | 79.7 | 20.3 | 0.3 | -0.5 | 276 | 1.8 |
| *ND6* | 33.3 | 16.8 | 6.0 | 43.9 | 77.2 | 22.8 | -0.1 | -0.5 | 483 | 3.2 |
| *CYTB* | 32.0 | 18.2 | 11.0 | 38.8 | 70.8 | 29.2 | -0.1 | -0.2 | 1,141 | 7.6 |
| *ND1* | 46.4 | 16.8 | 11.1 | 25.8 | 72.2 | 27.9 | 0.3 | -0.2 | 912 | 6.1 |
| 16s rRNA | 41.5 | 16.0 | 6.6 | 35.9 | 77.4 | 22.6 | 0.1 | -0.4 | 1,134 | 7.6 |
| 12s rRNA | 40.1 | 16.1 | 8.0 | 35.8 | 75.9 | 24.1 | 0.1 | -0.3 | 758 | 5.1 |
| *ND2* | 36.0 | 16.3 | 8.0 | 39.7 | 75.7 | 24.3 | 0.0 | -0.3 | 970 | 6.5 |
| PCGs | 38.6 | 17.1 | 9.9 | 34.4 | 73.0 | 27.0 | 0.1 | -0.3 | 10,798 | 72.1 |
| tRNAs | 39.6 | 14.9 | 10.3 | 35.3 | 74.9 | 25.1 | 0.1 | -0.2 | 1386 | 9.3 |
| rRNAs | 41.0 | 16.0 | 7.2 | 35.8 | 76.8 | 23.2 | 0.1 | -0.4 | 1892 | 12.6 |
| AT-rich region | 39.0 | 9.0 | 4.9 | 47.1 | 86.1 | 13.9 | -0.1 | -0.3 | 902 | 6.0 |
| Complete mtDNA | 39.0 | 16.3 | 9.3 | 35.4 | 74.4 | 25.6 | 0.0 | -0.3 | 14978 | 100.0 |
|  |  |  |  |  |  |  |  |  |  |  |
| **Tanzania** | | | | | | | | | | |
| **Region** | **A%** | **C%** | **G%** | **T%** | **A+T%** | **G+C%** | **AT-skew** | **GC-skew** | **Size (bp)** | **Size (%)** |
| *COI* | 32.4 | 17.7 | 13.2 | 36.7 | 69.1 | 30.9 | -0.1 | -0.1 | 1,530 | 10.2 |
| *COII* | 34.8 | 17.6 | 10.4 | 37.2 | 72.0 | 28.0 | 0.0 | -0.3 | 664 | 4.4 |
| *ATP8* | 35.9 | 20.9 | 5.2 | 38.6 | 74.5 | 26.1 | 0.0 | -0.6 | 153 | 1.0 |
| *ATP6* | 36.6 | 17.0 | 8.7 | 37.6 | 74.2 | 25.8 | 0.0 | -0.3 | 675 | 4.5 |
| *COIII* | 36.5 | 15.7 | 10.7 | 37.0 | 73.5 | 26.4 | 0.0 | -0.2 | 783 | 5.2 |
| *ND3* | 38.1 | 13.5 | 8.0 | 40.4 | 78.5 | 21.5 | 0.0 | -0.3 | 349 | 2.3 |
| *ND5* | 43.9 | 17.0 | 10.2 | 28.9 | 72.8 | 27.2 | 0.2 | -0.3 | 1,618 | 10.8 |
| *ND4* | 46.2 | 18.0 | 8.7 | 27.1 | 73.3 | 26.7 | 0.3 | -0.3 | 1,244 | 8.3 |
| *ND4L* | 50.4 | 14.9 | 5.4 | 29.3 | 79.7 | 20.3 | 0.3 | -0.5 | 276 | 1.8 |
| *ND6* | 33.3 | 16.8 | 6.0 | 43.9 | 77.2 | 22.8 | -0.1 | -0.5 | 483 | 3.2 |
| *CYTB* | 32.0 | 18.2 | 11.0 | 38.8 | 70.8 | 29.2 | -0.1 | -0.2 | 1,141 | 7.6 |
| *ND1* | 46.4 | 16.8 | 11.1 | 25.8 | 72.2 | 27.9 | 0.3 | -0.2 | 912 | 6.1 |
| 16s rRNA | 41.5 | 16.0 | 6.6 | 35.9 | 77.4 | 22.6 | 0.1 | -0.4 | 1,134 | 7.6 |
| 12s rRNA | 40.1 | 16.1 | 8.0 | 35.8 | 75.9 | 24.1 | 0.1 | -0.3 | 758 | 5.1 |
| *ND2* | 36.0 | 16.3 | 8.0 | 39.7 | 75.7 | 24.3 | 0.0 | -0.3 | 970 | 6.5 |
| **PCGs** | **38.6** | **17.1** | **9.9** | **34.4** | **73.0** | **27.0** | **0.1** | **-0.3** | **10,798** | **72.1** |
| tRNAs | 39.7 | 14.9 | 10.2 | 35.3 | 75.0 | 25.1 | 0.1 | -0.2 | 1482 | 9.9 |
| rRNAs | 41.0 | 16.0 | 7.2 | 35.8 | 76.8 | 23.2 | 0.1 | -0.4 | 1892 | 12.6 |
| AT-rich region | 39.1 | 9.3 | 4.8 | 46.8 | 85.9 | 14.1 | -0.1 | -0.3 | 902 | 6.0 |
| **Complete mtDNA** | **39.0** | **16.3** | **9.3** | **35.4** | **74.4** | **25.6** | **0.0** | **-0.3** | **14978** | **100.0** |
|  |  |  |  |  |  |  |  |  |  |  |
| **USA** | | | | | | | | | | |
| **Region** | **A%** | **C%** | **G%** | **T%** | **A+T%** | **G+C%** | **AT-skew** | **GC-skew** | **Size (bp)** | **Size (%)** |
| *COI* | 32.5 | 17.6 | 13.1 | 36.7 | 69.2 | 30.7 | -0.1 | -0.1 | 1,530 | 10.2 |
| *COII* | 34.6 | 17.3 | 10.4 | 37.7 | 72.3 | 27.7 | 0.0 | -0.2 | 664 | 4.4 |
| *ATP8* | 35.9 | 20.3 | 5.2 | 38.6 | 74.5 | 25.5 | 0.0 | -0.6 | 153 | 1.0 |
| *ATP6* | 36.9 | 17.0 | 8.4 | 37.6 | 74.5 | 25.5 | 0.0 | -0.3 | 675 | 4.5 |
| *COIII* | 36.4 | 15.7 | 10.9 | 37.0 | 73.4 | 26.6 | 0.0 | -0.2 | 783 | 5.2 |
| *ND3* | 38.1 | 13.5 | 8.0 | 40.4 | 78.5 | 21.5 | 0.0 | -0.3 | 349 | 2.3 |
| *ND5* | 44.0 | 16.9 | 10.1 | 28.9 | 72.9 | 27.1 | 0.2 | -0.3 | 1,618 | 10.8 |
| *ND4* | 46.1 | 18.1 | 8.8 | 27.0 | 73.1 | 26.9 | 0.3 | -0.3 | 1,244 | 8.3 |
| *ND4L* | 50.4 | 14.9 | 5.4 | 29.3 | 79.7 | 20.3 | 0.3 | -0.5 | 276 | 1.8 |
| *ND6* | 33.3 | 16.6 | 6.0 | 44.1 | 77.4 | 22.6 | -0.1 | -0.5 | 483 | 3.2 |
| *CYTB* | 31.8 | 18.3 | 11.0 | 38.8 | 70.6 | 29.4 | -0.1 | -0.2 | 1,141 | 7.6 |
| *ND1* | 46.4 | 16.7 | 11.2 | 25.8 | 72.2 | 27.9 | 0.3 | -0.2 | 912 | 6.1 |
| 16s rRNA | 41.7 | 15.8 | 6.4 | 36.0 | 77.7 | 22.3 | 0.1 | -0.4 | 1,134 | 7.6 |
| 12s rRNA | 40.1 | 16.0 | 8.0 | 35.9 | 76.0 | 24.0 | 0.1 | -0.3 | 758 | 5.1 |
| *ND2* | 35.9 | 16.2 | 8.1 | 39.8 | 75.7 | 24.3 | -0.1 | -0.3 | 970 | 6.5 |
| **PCGs** | **38.3** | **17.0** | **10.1** | **34.6** | **72.9** | **27.1** | **0.1** | **-0.3** | **10,798** | **72.1** |
| tRNAs | 39.6 | 14.8 | 10.3 | 35.3 | 74.9 | 25.1 | 0.1 | -0.2 | 1386 | 9.3 |
| rRNAs | 41.1 | 15.9 | 7.1 | 36.0 | 77.1 | 23.0 | 0.1 | -0.4 | 1892 | 12.6 |
| AT-rich region | 39.1 | 9.0 | 5.0 | 46.1 | 85.2 | 14.0 | -0.1 | -0.3 | 902 | 6.0 |
| **Complete mtDNA** | **39.0** | **16.3** | **9.3** | **35.4** | **74.4** | **25.6** | **0.0** | **-0.3** | **14978** | **100.0** |

**Table S8.** Pairwise comparison between two groups of *Diaphorina citri* specimens collected in China/Kenya/Tanzania (Group 1) and Ethiopia/USA (Group 2) given as the total number of single nucleotide polymorphisms (SNPs) across the complete mitogenomes, and non-synonymous amino acid substitutions (NS) in the 13 protein-coding genes.

| **Gene** | **Polymorphism type** | **Nucleotide substitution** | **Codon change** | **Amino acid substitution** |
| --- | --- | --- | --- | --- |
| COX1 |  |  |  |  |
|  | SNP (transition) | G -> A | CCG -> CCA | Synonymous |
|  | SNP (transition) | G -> A | TGG -> TGA | Synonymous |
|  | SNP (transition) | C -> T | CTA -> TTA | Synonymous |
|  | SNP (transition) | A -> G | ACT -> GCT | Non-synonymous (T -> A) |
|  | SNP (transversion) | C -> A | CAC -> CAA | Non-synonymous (H -> Q) |
|  | SNP (transition) | G -> A | TGG -> TGA | Synonymous |
| COX2 |  |  |  |  |
|  | SNP (transversion) | A -> T | ATA -> TTA | Non-synonymous (M -> L) |
|  | SNP (transition) | C -> T | ACC -> ACT | Synonymous |
|  | SNP (transition) | C -> T | TAC -> TAT | Synonymous |
| ATP8 |  |  |  |  |
|  | SNP (transition) | C -> T | ATC -> ATT | Synonymous |
|  | SNP (transversion) | T -> G | TAT -> GAT | Non-synonymous (Y -> D) |
| ATP6 |  |  |  |  |
|  | SNP (transition) | G -> A | TGG -> TGA | Synonymous |
|  | SNP (transition) | G -> A | TCG -> TCA | Synonymous |
| COX3 |  |  |  |  |
|  | SNP (transition) | A -> G | TCA -> TCG | Synonymous |
| ND3 |  |  |  |  |
|  | SNP (transition) | C -> T | CTA -> TTA | Synonymous |
|  | SNP (transition) | T -> C | AAT -> AAC | Synonymous |
| ND5 |  |  |  |  |
|  | SNP (transition) | T -> C | AAA -> AAG | Synonymous |
|  | SNP (transition) | C -> T | ATG -> ATA | Synonymous |
|  | SNP (transition) | C -> T | GTG -> GTA | Synonymous |
|  | SNP (transition) | T -> C | GGA -> GGG | Synonymous |
|  | SNP (transition) | G -> A | CTT -> TTT | Non-synonymous (L -> F) |
|  | SNP (transition) | C -> T | GTA -> ATA | Non-synonymous (V -> M) |
| ND4 |  |  |  |  |
|  | SNP (transition) | A -> G | CCT -> CCC | Synonymous |
|  | SNP (transition) | G -> A | CTA -> TTA | Synonymous |
|  | SNP (transition) | T -> C | ATA -> ATG | Synonymous |
|  | SNP (transition) | A -> G | TTT -> TTC | Synonymous |
|  | SNP (transition) | A -> G | ATT -> ATC | Synonymous |
|  | SNP (transition) | G -> A | CAC -> CAT | Synonymous |
|  | SNP (transition) | A -> G | GGT -> GGC | Synonymous |
| ND6 |  |  |  |  |
|  | SNP (transition) | T -> C | GTT -> GTC | Synonymous |
|  | Substitution of two bases | CTC -> TTT | CTC -> TTT | Non-synonymous (L -> F) |
| CYTB |  |  |  |  |
|  | SNP (transition) | A -> G | GGA -> GGG | Synonymous |
|  | SNP (transversion) | A -> T | GGA -> GGT | Synonymous |
|  | SNP (transition) | T -> C | CCT -> CCC | Synonymous |
| ND1 |  |  |  |  |
|  | SNP (transition) | C -> T | GGA -> AGA | Non-synonymous (G -> S) |
|  | SNP (transition) | T -> C | GAA -> GAG | Synonymous |
|  | SNP (transition) | A -> G | ATT -> ATC | Synonymous |
|  | SNP (transversion) | C -> A | GCT -> TCT | Non-synonymous (A -> S) |
| ND2 |  |  |  |  |
|  | SNP (transition) | A -> G | ATT -> GTT | Non-synonymous (I -> V) |
|  | SNP (transition) | C -> T | TCC -> TTC | Non-synonymous (S -> F) |

**Table S9.** Genetic distances of *Diaphorina citri* from China, Ethiopia, Kenya, Tanzania and USA from this study (in bold) and other countries available on GenBank (n =18), based on an 874 bp alignment of *COI*. Distances were calculated as percentage of pairwise distances (p-distances). Standard error estimates are shown above the diagonal.

|  |  | Nucleotide pairwise comparison (% p-distances) | | | | | | | | | | | | | | | | | | | | | | |
| --- | --- | --- | --- | --- | --- | --- | --- | --- | --- | --- | --- | --- | --- | --- | --- | --- | --- | --- | --- | --- | --- | --- | --- | --- |
|  |  | 1 | 2 | 3 | 4 | 5 | 6 | 7 | 8 | 9 | 10 | 11 | 12 | 13 | 14 | 15 | 16 | 17 | 18 | 19 | 20 | 21 | 22 | 23 |
| 1 | China | - | 0.00 | 0.01 | 0.00 | 0.00 | 0.00 | 0.00 | 0.00 | 0.00 | 0.00 | 0.00 | 0.00 | 0.00 | 0.00 | 0.00 | 0.00 | 0.00 | 0.08 | 0.08 | 0.08 | 0.08 | 0.08 | 0.08 |
| 2 | Argentina | 0.24 | - | 0.00 | 0.00 | 0.00 | 0.00 | 0.00 | 0.00 | 0.00 | 0.00 | 0.00 | 0.00 | 0.00 | 0.00 | 0.00 | 0.00 | 0.00 | 0.08 | 0.08 | 0.08 | 0.08 | 0.08 | 0.08 |
| 3 | Brazil | 1.20 | 0.96 | - | 0.00 | 0.00 | 0.00 | 0.00 | 0.01 | 0.00 | 0.00 | 0.00 | 0.00 | 0.01 | 0.00 | 0.00 | 0.00 | 0.00 | 0.08 | 0.08 | 0.08 | 0.08 | 0.08 | 0.08 |
| 4 | Iran | 0.72 | 0.48 | 0.96 | - | 0.00 | 0.00 | 0.00 | 0.00 | 0.00 | 0.00 | 0.00 | 0.00 | 0.00 | 0.00 | 0.00 | 0.00 | 0.00 | 0.08 | 0.08 | 0.08 | 0.08 | 0.08 | 0.08 |
| 5 | Malaysia | 0.24 | 0.00 | 0.96 | 0.48 | - | 0.00 | 0.00 | 0.00 | 0.00 | 0.00 | 0.00 | 0.00 | 0.00 | 0.00 | 0.00 | 0.00 | 0.00 | 0.08 | 0.08 | 0.08 | 0.08 | 0.08 | 0.08 |
| 6 | Mexico | 0.72 | 0.48 | 0.96 | 0.00 | 0.48 | - | 0.00 | 0.00 | 0.00 | 0.00 | 0.00 | 0.00 | 0.00 | 0.00 | 0.00 | 0.00 | 0.00 | 0.08 | 0.08 | 0.08 | 0.08 | 0.08 | 0.08 |
| 7 | Pakistan | 0.72 | 0.48 | 0.96 | 0.00 | 0.48 | 0.00 | - | 0.00 | 0.00 | 0.00 | 0.00 | 0.00 | 0.00 | 0.00 | 0.00 | 0.00 | 0.00 | 0.08 | 0.08 | 0.08 | 0.08 | 0.08 | 0.08 |
| 8 | Puerto Rico | 0.48 | 0.24 | 1.20 | 0.24 | 0.24 | 0.24 | 0.24 | - | 0.00 | 0.00 | 0.00 | 0.00 | 0.00 | 0.00 | 0.00 | 0.00 | 0.00 | 0.08 | 0.08 | 0.08 | 0.08 | 0.08 | 0.08 |
| 9 | Thailand | 0.24 | 0.00 | 0.96 | 0.48 | 0.00 | 0.48 | 0.48 | 0.24 | - | 0.00 | 0.00 | 0.00 | 0.00 | 0.00 | 0.00 | 0.00 | 0.00 | 0.08 | 0.08 | 0.08 | 0.08 | 0.08 | 0.08 |
| 10 | USA | 0.72 | 0.48 | 0.96 | 0.00 | 0.48 | 0.00 | 0.00 | 0.24 | 0.48 | - | 0.00 | 0.00 | 0.00 | 0.00 | 0.00 | 0.00 | 0.00 | 0.08 | 0.08 | 0.08 | 0.08 | 0.08 | 0.08 |
| 11 | Cambodia | 0.24 | 0.00 | 0.96 | 0.48 | 0.00 | 0.48 | 0.48 | 0.24 | 0.00 | 0.48 | - | 0.00 | 0.00 | 0.00 | 0.00 | 0.00 | 0.00 | 0.08 | 0.08 | 0.08 | 0.08 | 0.08 | 0.08 |
| 12 | **China** | 0.24 | 0.00 | 0.96 | 0.48 | 0.00 | 0.48 | 0.48 | 0.24 | 0.00 | 0.48 | 0.00 | - | 0.00 | 0.00 | 0.00 | 0.00 | 0.00 | 0.08 | 0.08 | 0.08 | 0.08 | 0.08 | 0.08 |
| 13 | **Ethiopia** | 0.96 | 0.72 | 1.20 | 0.24 | 0.72 | 0.24 | 0.24 | 0.48 | 0.72 | 0.24 | 0.72 | 0.72 | - | 0.00 | 0.00 | 0.00 | 0.00 | 0.08 | 0.08 | 0.08 | 0.08 | 0.08 | 0.08 |
| 14 | **Kenya** | 0.24 | 0.00 | 0.96 | 0.48 | 0.00 | 0.48 | 0.48 | 0.24 | 0.00 | 0.48 | 0.00 | 0.00 | 0.72 | - | 0.00 | 0.00 | 0.00 | 0.08 | 0.08 | 0.08 | 0.08 | 0.08 | 0.08 |
| 15 | **Tanzania** | 0.24 | 0.00 | 0.96 | 0.48 | 0.00 | 0.48 | 0.48 | 0.24 | 0.00 | 0.48 | 0.00 | 0.00 | 0.72 | 0.00 | - | 0.00 | 0.00 | 0.08 | 0.08 | 0.08 | 0.08 | 0.08 | 0.08 |
| 16 | **USA** | 0.72 | 0.48 | 0.96 | 0.00 | 0.48 | 0.00 | 0.00 | 0.24 | 0.48 | 0.00 | 0.48 | 0.48 | 0.24 | 0.48 | 0.48 | - | 0.00 | 0.08 | 0.08 | 0.08 | 0.08 | 0.08 | 0.08 |
| 17 | Taiwan | 0.24 | 0.00 | 0.96 | 0.48 | 0.00 | 0.48 | 0.48 | 0.24 | 0.00 | 0.48 | 0.00 | 0.00 | 0.72 | 0.00 | 0.00 | 0.48 | - | 0.08 | 0.08 | 0.08 | 0.08 | 0.08 | 0.08 |
| 18 | Saudi Arabia | 7.90 | 7.91 | 8.08 | 7.82 | 7.91 | 7.82 | 7.82 | 7.81 | 7.91 | 7.82 | 7.91 | 7.91 | 7.76 | 7.91 | 7.91 | 7.82 | 7.91 | - | 0.00 | 0.00 | 0.00 | 0.00 | 0.00 |
| 19 | Guadeloupe | 7.77 | 7.78 | 7.95 | 7.69 | 7.78 | 7.69 | 7.69 | 7.69 | 7.78 | 7.69 | 7.78 | 7.78 | 7.64 | 7.78 | 7.78 | 7.69 | 7.78 | 0.72 | - | 0.00 | 0.00 | 0.00 | 0.00 |
| 20 | India | 7.90 | 7.91 | 8.08 | 7.82 | 7.91 | 7.82 | 7.82 | 7.81 | 7.91 | 7.82 | 7.91 | 7.91 | 7.76 | 7.91 | 7.91 | 7.82 | 7.91 | 0.00 | 0.72 | - | 0.00 | 0.00 | 0.00 |
| 21 | Indonesia | 7.90 | 7.91 | 8.08 | 7.82 | 7.91 | 7.82 | 7.82 | 7.81 | 7.91 | 7.82 | 7.91 | 7.91 | 7.76 | 7.91 | 7.91 | 7.82 | 7.91 | 0.00 | 0.72 | 0.00 | - | 0.00 | 0.00 |
| 22 | Mauritius | 7.72 | 7.73 | 7.89 | 7.64 | 7.73 | 7.64 | 7.64 | 7.64 | 7.73 | 7.64 | 7.73 | 7.73 | 7.58 | 7.73 | 7.73 | 7.64 | 7.73 | 0.48 | 0.72 | 0.48 | 0.48 | - | 0.00 |
| 23 | Reunion | 7.72 | 7.73 | 7.89 | 7.64 | 7.73 | 7.64 | 7.64 | 7.64 | 7.73 | 7.64 | 7.73 | 7.73 | 7.58 | 7.73 | 7.73 | 7.64 | 7.73 | 0.48 | 0.72 | 0.48 | 0.48 | 0.00 | - |
